# Medicinal Plants for Child Mental Health: Clinical Insights, Active Compounds, and Perspectives for Rational Use

**DOI:** 10.3390/children12091142

**Published:** 2025-08-28

**Authors:** Giovanna Rigillo, Joan M. C. Blom, Arianna Cocchi, Valentina Martinucci, Francesca Favaro, Giulia Baini, Giorgio Cappellucci, Fabio Tascedda, Marco Biagi

**Affiliations:** 1 Department of Biomedical, Metabolic and Neural Sciences, University of Modena and Reggio Emilia, 41125 Modena, Italy; giovanna.rigillo@unimore.it (G.R.); joan.blom@unimore.it (J.M.C.B.); 2 Pediatrica S.r.l., 57121 Livorno, Italy; rd02@pediatricaspecialist.it (A.C.); rd@pediatrica.it (V.M.); rd03@pediatricaspecialist.it (F.F.); 3 Department of Physical Sciences, Earth and Environment, University of Siena, 53100 Siena, Italy; giulia.baini2@unisi.it (G.B.); giorgi.cappellucci@unisi.it (G.C.); 4 Department of Life Sciences, University of Modena and Reggio Emilia, 41125 Modena, Italy; fabio.tascedda@unimore.it; 5 CIB, Consorzio Interuniversitario Biotecnologie, 34127 Trieste, Italy; 6 Department of Food and Drug, University of Parma, 43124 Parma, Italy

**Keywords:** botanicals, children, clinical study, mental health, target prediction, phytotherapy

## Abstract

**Highlights:**

**What are the main findings?**

- Twenty-nine clinical trials evaluated herbal products in children and adolescents.
- Outcomes measured in clinical trials support the safety and preliminary efficacy of selected herbal products for mental well-being.
- Bioinformatic analysis revealed the multitarget activity of herbal products with regard to neuroprotection and neurotransmission.

**What is the implication of the main finding?**

- Herbal medicines may represent safe complementary tools in pediatric mental health care.
- Clinical use requires standardized extracts and medical supervision.
- Further studies are essential to clarify dosing, long-term safety, and integrative strategies.

**Abstract:**

Background/Objectives: Anxiety, agitation, and mood disturbances are increasingly common among children and adolescents. Given the limitations of conventional pharmacological treatments in the pediatric population, particularly for subthreshold or mild conditions, interest in complementary approaches such as phytotherapy is growing. This review aims to critically evaluate the clinical evidence supporting the use of herbal medicines and botanical food supplements for mental health symptoms in youths and to explore the pharmacological basis of their activity. Methods: A systematic search was conducted across main databases for clinical trials involving herbal products for psychologically related symptoms in children and adolescents. Eligible studies included those using registered herbal medicines, as well as authorized food supplements, that evaluated behavioral or cognitive outcomes. In addition, bioinformatic analyses were performed on selected phytocompounds to predict their molecular targets. Results: Twenty-nine clinical trials were identified, including eighteen targeting pathological conditions (notably attention deficit/hyperactivity disorder (ADHD), anxiety, and depression) and eleven addressing borderline symptoms such as nervous agitation, restlessness, or sleep disturbances. Herbal products showing clinical promise include *Bacopa monnieri* (L.) Wettst., *Crocus sativus* L., *Ginkgo biloba* L., *Hypericum perforatum* L., *Lavandula angustifolia* Mill., *Melissa officinalis* L., *Panax ginseng* C.A. Meyer, *Passiflora incarnata* L., *Pinus pinaster* Aiton, *Valeriana officinalis* L., and *Withania somnifera* (L.) Dunal. Bioinformatic predictions revealed polypharmacological activity profiles involving neuroinflammatory, neuroprotective, and neurotransmitter-related pathways. Conclusions: This review highlights both the potential and the current limitations of herbal products in pediatric mental health care. Evidence supports their use for selected indications, provided that standardized preparations and clinical oversight are ensured. Further research is essential, particularly to inform dosing, safety, and integrative care strategies.

## 1. Introduction

Childhood and adolescence represent critical windows of neurodevelopment during which the brain is especially vulnerable to environmental, emotional, and physiological influences [1,2]. In these developmental stages, the systems responsible for emotional, cognitive, and behavioral regulation are still maturing, rendering young individuals particularly susceptible to mental health disturbances. Excessive anxiety, agitation, hyperactivity, inattention, sleep disorders, and mood instability are frequently reported, and, even as subclinical conditions, can significantly impair psychosocial functioning, academic performance, and overall well-being [3,4,5]. Early-life exposure to pain, stress, or trauma can profoundly alter neurodevelopmental trajectories, disrupting stress-response systems and emotional regulation mechanisms [6]. These adverse experiences have been linked not only to the emergence of anxiety and hyperactivity, but also to more complex psychiatric conditions such as attention deficit/hyperactivity disorder (ADHD) and depression [7]. Understanding the developmental context in which these disorders arise and addressing these symptoms early is therefore crucial to improving the diagnosis, treatment, and prevention of long-term psychological and functional impairments.

Among psychiatric conditions, anxiety disorders are the most prevalent in the pediatric population, often manifesting early and potentially persisting into adulthood. Global data show a 52% increase in anxiety disorders among individuals aged 10–24 years between 1990 and 2021, with around 16.7 million new cases reported in 2021 alone, and a higher prevalence in females [3,5,8,9,10]. Social anxiety disorder, for example, affects approximately 4.7% of children and 8.3% of adolescents. While some symptoms may spontaneously remit, many youth develop chronic conditions or comorbid disorders such as depression [9], suicidal behavior [11,12], or substance use disorders [13], as well as academic difficulties [14], later in life.

The Diagnostic and Statistical Manual of Mental Disorders, Fifth Edition (DSM-5) [15] introduced key revisions to the classification of anxiety disorders in youth (Table S1). Separation anxiety disorder and selective mutism are now recognized regardless of the age of onset, reflecting their persistence beyond childhood. Other changes include the removal of the requirement for adults to recognize their fears as excessive and the standardization of a six-month minimum duration criterion across all age groups [16].

Symptoms of anxiety may present differently across developmental stages. For example, children with separation anxiety may exhibit clinging or school refusal, whereas adolescents may develop social avoidance and performance anxiety [14,17]. Sleep disturbances are also common in youth with anxiety disorders and may act both as symptoms and as contributing factors. Research suggests that the majority of anxious youth experience significant sleep-related problems, which can further impair daytime functioning and emotional regulation [4,18].

Emerging evidence highlights how sleep problems during developmental transitions can predict both internalizing symptoms (such as anxiety and depression) and externalizing symptoms (such as aggression) [4]. Anxiety and ADHD, which are often co-occurring, create a complex clinical picture, with overlapping features such as restlessness and impaired concentration, which complicate diagnosis and management. It is estimated that up to 50% of individuals with ADHD will experience an anxiety disorder during their lifetime [19,20,21]. Comorbidity exacerbates functional impairment, increases executive dysfunction, and raises the risk of depression and substance abuse.

Assessment strategies must integrate multiple sources, such as self-report, caregiver input, and teacher observations, due to frequent discrepancies [15,22,23]. Management typically follows a stepped approach combining cognitive behavioral therapy (CBT) [24,25], family involvement [26,27,28], and personalized support targeting both psychological and biological dimensions. Adjunctive strategies include mindfulness, exercise, and dietary interventions [29,30], while pharmacological options, such as selective serotonin reuptake inhibitors (SSRIs), are used in more severe cases, albeit with caution due to developmental concerns [31].

In this landscape, herbal products may represent a complementary strategy, especially in cases where mild symptoms do not warrant pharmacological treatment with synthetic drugs or when parents and clinicians seek alternatives for which the safety profile is a priority [32]. In fact, herbal products occupy a unique niche, as they are used both in the form of registered medicinal products and as botanical food supplements intended to support mental well-being [33].

The European Medicines Agency (EMA) recognizes the traditional or well-established use of 14 medicinal plants for mental stress and mood disorders and/or sleep disturbances, including anxiolytics (e.g., *Valeriana officinalis* L., *Passiflora incarnata* L., *Melissa officinalis* L., *Lavandula angustifolia* Mill.), adaptogens (e.g., *Rhodiola rosea* L.), and mood enhancers (e.g., *Hypericum perforatum* L.) (the complete list is in Table S2) [34]. Many of these species are also included in the lists of botanicals authorized as food supplements both in European Union (EU) countries and in other countries; in fact, the number of species used in food supplements with a health claim related to mental well-being is very large, including more than 50 in Italy alone that are authorized by the Italian Ministry of Health [35]. Of note, some essential oils derived from medicinal plants, either pharmaceutical-grade or in the form of natural fragrances, are also used by inhalation to produce health benefits through the olfactory response. The regulatory classification of essential oils is challenging, as they may be used for both self-care and medically supervised treatment. Among them, lavender oil (*L. angustifolia*) is the most extensively documented.

Depending on their regulatory status, herbal products must comply with distinct manufacturing and quality standards, and their intended use and target populations differ accordingly. Botanical food supplements are designed to support general well-being; safety is their primary attribute, and they are broadly applicable, including in pediatric populations, although dosage restrictions and specific warnings may apply. In contrast, herbal medicines are developed for defined therapeutic indications, with efficacy demonstrated through traditional or well-established use, and their side effects and precautions explicitly reported. For this reason, herbal medicines acting on the central nervous system (CNS) are generally recommended for children over 12 years of age, or in certain cases restricted to adult use only.

This review aims to provide a critical and integrative overview of current clinical evidence on the use of medicinal plants for managing mental health symptoms in pediatric and adolescent populations. Specifically, it focuses on

1. selecting and analyzing existing clinical trials involving plant-based interventions in this age group; and
2. exploring the molecular mechanisms of the most promising phytocomplexes by integrating emerging evidence with bioinformatic analyses.

By synthesizing current knowledge, this work seeks to offer a practical tool for clinicians and researchers, promoting evidence-based integration of phytotherapy into treatment planning for children and adolescents facing mental disturbances.

## 2. Methods

### 2.1. Search Strategy

This systematic review was conducted following the Preferred Reporting Items for Systematic Reviews and Meta-Analyses (PRISMA) 2020 statement [36].

Six conventional electronic databases (PubMed, Scopus, Web of Science, Embase, clinicaltrials.gov, and Google Scholar) and available full-text articles found in ResearchGate were systematically searched for studies related to mental health conditions and related symptoms in children, adolescents, and youth. The literature search strategy consisted of the following terms: [“phytotherapy” OR “herbal” OR “botanicals” OR “medicinal plants”] and [anxiety OR agitation OR hyperactivity OR stress OR sleep OR mood] and [children OR pediatric OR adolescents OR youth]. Studies were searched from inception to 15 June 2025, without date restrictions. Titles and abstracts were independently screened, and the data were extracted by two of the authors (GR and MB), with discrepancies resolved through discussion. Duplicates were removed prior to screening.

### 2.2. Eligibility Criteria

In order to be included in the systematic review, a study had to meet the following eligibility criteria: (a) designed as a randomized controlled trial (RCT) (blind, double-blind, cross-over, or open-label) or observational study; (b) inclusion of children or adolescents only; (c) inclusion of subjects with pathological symptoms or subthreshold conditions related to mental health, namely anxiety, hyperactivity, agitation, inattention, mood disorders, cognitive impairments, and emotional dysregulation; (d) children with mental health conditions assessed according to recognized diagnostic systems or validated clinical instruments; (e) subjects treated with herbal products orally (or through inhalation for essential oils) and compared to either placebo or any approved drug (with no limitation in the dosing scheme or duration of intervention) or nothing; and (f) outcomes assessed through validated and well-established measures, including cognitive performance tests, psychiatric rating scales, symptom rating scales, or biological parameters, before and after the intervention. Authorized herbal food supplements and registered herbal medicines were considered as treatments. Traditional Chinese Medicine (TCM) formulations, as well as other traditional products approved only outside Europe or the United States, were not considered.

For studies investigating the inhalation of essential oils, due to the high heterogeneity of the field and the frequent lack of clear methodological descriptions, only RCTs in which the intervention was clearly identifiable from the title and abstract and consisted of a single, well-defined treatment were included.

In the selected studies, herbal products were used either as approved pediatric products or off-label, with parental consent and ethical approval.

Papers not in English, review articles, commentaries, letters, theses, animal studies, in vitro studies, and conference abstracts were excluded. After the first-round search, the retrieved articles were classified as

- clinical trials targeting ADHD and other severe psychiatric conditions; or
- clinical trials targeting mild symptoms and non-pathological conditions, such as mild anxiety, mental stress, mild mood disorders, and temporary sleep disturbances.

### 2.3. Risk-of-Bias Assessment and Grading of Recommendations, Assessment, Development, and Evaluation

Risk of bias was independently assessed by two reviewers (GR and MB). For randomized controlled trials, the Cochrane Risk of Bias tool (RoB2) was used, while for non-randomized studies the ROBINS-I tool was applied. Discrepancies were resolved through discussion.

Given the heterogeneity of the interventions (multiple herbal products used and different regulatory status) and outcomes (different mental health domains), formal Grading of Recommendations, Assessment, Development, and Evaluation (GRADE) was not feasible for individual plants. Instead, a semiquantitative evidence evaluation inspired by the GRADE framework was applied per mental health domain or pathological condition. The main GRADE domains––risk of bias, inconsistency, indirectness, imprecision, and publication bias––were considered.

Based on these domains, the strength of evidence for each mental health outcome was classified qualitatively as “very low”, “low”, or “moderate” (none of the botanical interventions reached a “high” level of certainty of evidence). Risk of bias and GRADE were applied at the study level.

### 2.4. Bioinformatic Analysis

According to Rigillo et al. (2025) [37], computational analysis of the pharmacokinetic characteristics of known active components of selected medicinal plants was performed using the SwissADME^®^ web tool. Target prediction was performed using the GeneCards suite and SuperPred tools, which combine chemical similarity and functionality and relevance scoring, as well as Similarity Ensemble Approach (SEA) and Swiss Target Prediction (STP), whose predictions are based on resemblance to ligands annotated and validated in reference databases.

## 3. Results

### 3.1. Search Results and Selection Process

**Figure 1:**
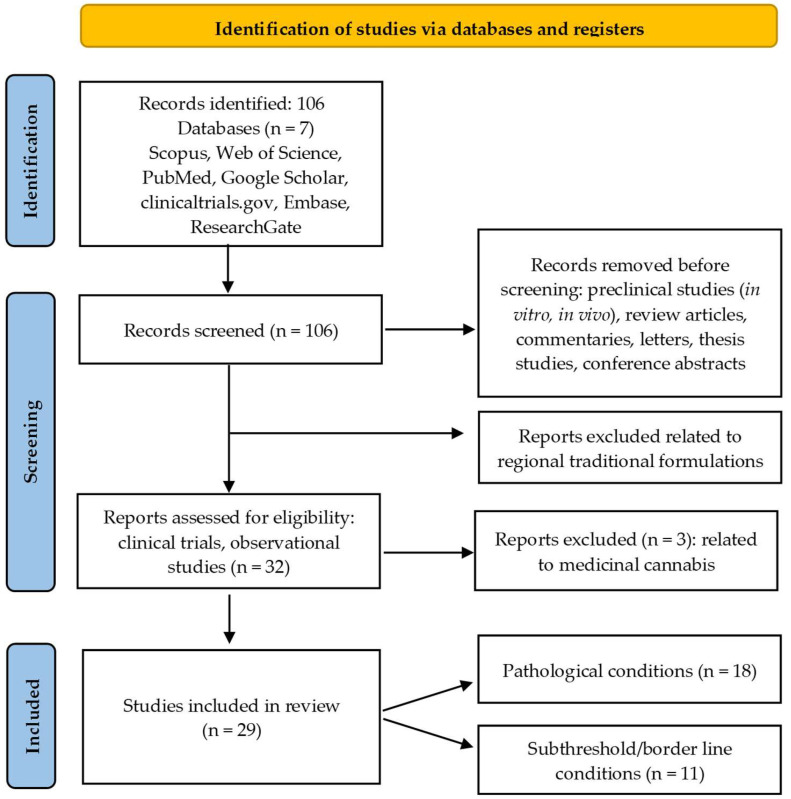
PRISMA flow diagram of the literature search, study selection, and eligibility process.

The PRISMA flow diagram shows the details of the selection process (Figure 1). A total of 106 papers were considered for critical analysis. The final selection consisted of 32 clinical trials, but 3 were rejected for being related to the use of medicinal cannabis, a topic which was out of the scope of this work. Eighteen clinical trials were related to pathological conditions: ADHD (16 papers), the autistic spectrum (1 paper), or major depressive symptoms (1 paper).

Eleven papers were related to subthreshold/borderline conditions, such as subthreshold ADHD (1), mild anxiety and depression (1), anxiety and psychological discomfort related to procedural pain (5), dyslexia (1), and nervous agitation and/or dyssomnia and/or inattention and hyperactivity (3).

### 3.2. Evidence from Clinical Studies

#### 3.2.1. Herbal Products for Management of Agitation, Dyssomnia, and Restlessness

Agitation, restlessness, and dyssomnia are prevalent issues in the pediatric populations, often stemming from a combination of behavioral, psychological, and physiological factors. Common contributors include inconsistent sleep routines, anxiety, ADHD, and environmental stressors. These disturbances can significantly impact a child’s cognitive development, emotional regulation, and overall well-being.

Some observational studies have investigated the efficacy of herbal products (Table 1) in reducing symptom scores, as assessed through validated scales. Müller and Klement (2006) conducted a multicentric observational study assessing a standardized medicinal combination of valerian (*Valeriana officinalis* L. root and rhizome) and lemon balm (*Melissa officinalis* L. leaves) dry extracts (Euvegal^®^) in children aged 6 to 11 years experiencing restlessness and dyssomnia [38]. The study found significant improvements in sleep quality and reductions in restlessness, recorded both by parents and investigators, with a favorable safety profile.

Further supporting these findings, Gromball et al. (2014). conducted a multicenter observational study of primary school children exhibiting hyperactivity, concentration difficulties, impulsiveness, and sleep disturbances [39]. Over a seven-week period, treatment with Sandrin^®^ (an earlier trade name of Euvegal^®^) led to significant improvements in these behavioral parameters 

Similarly, Trompetter et al. (2013) evaluated an herbal combination of St. John’s Wort (SJW) (*Hypericum perforatum* L. flowering aerial parts), valerian, and passionflower (*Passiflora incarnata* L. aerial parts) dry extracts (not pharmaceutical-grade) in children with nervous agitation, through an observational study [40]. The results indicated notable reductions in several agitation, anxiety and behavioral symptoms, and insomnia, suggesting the potential of this herbal triplet as a natural therapeutic option. No safety concerns were highlighted in the studies considered.

All the medicinal plants considered in these observational studies are listed in EMA monographs and recognized for their role in mental stress and mood disorders (Table S2); as food supplements, they are also specifically used to support mental well-being.

These studies collectively highlight the potential role of specific herbal formulations in managing pediatric agitation, restlessness, and sleep disturbances. However, the risk-of-bias assessment revealed serious concerns (high risk) and the level of evidence was rated as “very low” (Table S3), as no RCTs have been published, and further large-scale, long-term studies are warranted to confirm their efficacy and safety profiles.

#### 3.2.2. Herbal Products for Anxiety and Psychological Discomfort Related to Procedural Pain

Anxiety and distress related to procedural pain are frequent challenges in pediatric care, often resulting in increased pharmacological intervention and potential psychological trauma. While standard treatments remain essential, integrative strategies aimed at enhancing comfort and emotional well-being are gaining attention, particularly in cases of mild to moderate distress.

Interestingly, aromatherapy emerged as a promising non-pharmacological approach; all the studies retrieved in the pediatric population were related to the use of *L. angustifolia* (lavender) essential oil (in one case in combination with *Zingiber officinale* Roscoe rhizome essential oil) (Table 2), confirming the prevalence of this natural product in scientific aromatherapy.

Lavender oil is well known for its calming and anxiolytic properties and is included in EMA monographs (Table S2); however, in adults it is mostly used orally and as a bath additive. In contrast, several RCTs have shown that lavender inhalation could reduce procedural pain, anxiety, and physiological stress in children in several procedural contexts.

For dental procedures, including tooth extraction or dental treatment, lavender inhalation effectively reduces anticipatory anxiety and perceived pain, improving cooperation and the overall clinical experience; lavender oil also proved efficacious in affecting objective outcomes related to stress and anxiety, such as blood pressure, pulse rate, and cortisol level [41,42]. Supporting this effect, inhalation of lavender oil during dressing changes in pediatric burn patients reduced pain perception and stabilized vital signs (blood pressure, pulse, respiration) [43]. Postoperative lavender use after tonsillectomy decreased pain perception and consequently decreased analgesic requirements [44].

Moreover, in perioperative settings, combined lavender and ginger essential oil inhalation enhanced comfort and reduced postoperative nausea, addressing both psychological and somatic symptoms [45].

These findings support the adjunctive use of essential oil-based aromatherapy as a safe, non-invasive, and cost-effective complement to conventional pediatric care, contributing to a more positive healthcare experience for children.

The overall risk of bias across the five included RCTs ranged from “some concerns” in the two blinded studies to “high risk” in the three non-blinded trials using mainly subjective scales. According to GRADE, the quality of evidence was rated as “moderate” for objective outcomes such as cortisol level, number of respirations, blood pressure, pulse rate, and need for analgesics and “low” for subjective outcomes, primarily due small sample sizes and reliance on reported measures (Table S3).

#### 3.2.3. Medicinal Plant for Attention Deficit/Hyperactivity Disorder

ADHD is a prevalent neurodevelopmental disorder, typically manifesting in early childhood, and is characterized by persistent inattention, hyperactivity, and impulsivity. These symptoms can significantly impair academic performance, social functioning, and emotional regulation. Given the limitations and side effects of conventional pharmacological treatments, there is increasing interest in alternative and complementary approaches. Among these, several herbal products have shown potential in modulating ADHD symptoms, particularly in improving attention, reducing hyperactivity, and enhancing behavioral control [46,47].

The medicinal plants that emerged from studies were *Panax ginseng* C.A. Meyer roots (ginseng), *Pinus pinaster* Aiton bark (French maritime pine), *Withania somnifera* (L.) Dunal roots (withania or ashwagandha), *Bacopa monnieri* (L.) Wettst. aerial parts (bacopa), *Crocus sativus* L. stigma (saffron), SJW, *Ginkgo biloba* L. leaves (ginkgo), and the combination of valerian and lemon balm (Table 3).

In most studies, parents and/or assessors used validated ADHD rating scales (RSs) such as ADHD-RS-IV before and after the treatment to evaluate its efficacy. Three studies (two RCTs involving 190 children and one open-label trial) showed that ginseng, administered either as an herbal powder in accordance with Asian official pharmacopoeias or as a dry extract, improved attention and reduced hyperactivity in children with ADHD or subthreshold symptoms [48,49,50]. Although current clinical evidence is very limited, ginseng showed signals of safety and efficacy, warranting further investigation. Notably, ginseng demonstrated efficacy in subthreshold ADHD when combined with omega-3, which, in previous studies, showed only limited benefits regarding core ADHD symptoms when used alone [51].

Pycnogenol^®^ (Pyc), a standardized extract from *P. pinaster* bark that is rich in catechins, oligomeric procyanidins (OPC), and phenolic acids and authorized for the formulation of food supplements, has been evaluated in pediatric ADHD in three RCTs involving a small number of participants. Studies by Dvořáková et al. (2006, 2007) demonstrated that Pyc reduced oxidized glutathione (GSSG), increased reduced glutathione (GSH), and improved the GSH/GSSG ratio, key indicators of antioxidant capacity [52,53]. Elevated baseline catecholamines (dopamine, noradrenaline, adrenaline), often associated with oxidative stress, were also normalized post-treatment, suggesting modulation of both redox balance and neurochemical pathways. These biochemical effects exerted by Pyc correlated with behavioral improvements, particularly in the attention and hyperactivity domains. In fact, a four-week intervention with Pyc also yielded significant improvements in inattention and hyperactivity symptoms, as assessed by behavioral rating scales [54], and was well tolerated.

*W. somnifera* is a medicinal plant traditionally used for its beneficial effects on CNS disorders. Some recent clinical trials reported beneficial effects of withania preparations on CNS, endocrine, and cardiovascular function in adults [55,56]. However, only one document was found that focused on pediatric ADHD, namely an RCT conducted in 28 children with a ADHD diagnosis, which showed the potential of withania dry extract (not pharmaceutical grade) in reducing anxiety and social concerns [57].

*B. monnieri*, traditionally used to enhance memory and attention, has shown mixed results in ADHD populations. Some studies reported improvements in cognition, mood, and sleep quality in boys aged 6–14, although the effects on core ADHD symptoms remain inconclusive [58,59,60,61]. In detail, one RCT found that long-term bacopa supplementation had some effects on mood, cognition, and sleep quality, though social interaction and behavioral symptoms were unaffected [59]. On the other hand, in one small open-label study involving 31 children, bacopa was demonstrated to significantly reduce the majority of ADHD symptoms [61]. In both studies, standardized dry extracts authorized as ingredients for food supplements were used, and no safety concerns were recorded.

Saffron emerged as a promising therapeutic option for ADHD, owing to its neuroprotective and psychoactive properties [62,63,64,65,66,67,68]. Recent clinical trials demonstrated that short-term saffron supplementation in children with ADHD provided efficacy and tolerability comparable to methylphenidate (MPH), the standard pharmacological treatment, as reviewed in [69]. An RCT lasting six weeks reported similar changes in Teacher and Parent ADHD-RS-IV scores for saffron and MPH, with no differences in safety [70]. These findings have been supported by subsequent studies––one RCT and one non-randomized clinical trial [71,72]––including evidence that combining saffron with MPH may enhance treatment efficacy, suggesting potential for both monotherapy and adjunctive use in ADHD management. In clinical trials, saffron is mostly used as a powdered herbal substance or as dry extracts with a quantified content of crocins and, very often, of safranal.

Despite its well-established role as an antidepressant drug in adults, when used in the form of an extract not meeting pharmaceutical criteria, *H. perforatum* did not demonstrate efficacy in pediatric ADHD. An RCT evaluating its use reported no significant improvements in symptom scores [73].

*G. biloba* has been extensively studied for its neurocognitive and anti-ischemic benefits, as well as for its ability to regulate brain blood flow [74,75,76]. The most common target population for ginkgo is the elderly, especially for managing symptoms of mild cognitive impairment (MCI) and mild dementia. However, some clinical trials have been published focusing on children.

A double-blind RCT by Shakibaei et al. (2015) showed significant reductions in ADHD-RS-IV parent-rated inattention scores and total scores recorded after the use of an herbal medicine containing *G. biloba* standardized extract that were greater than those recorded with placebo [77]. A comparative RCT by Salehi et al. (2010) found that the same ginkgo preparation reduced some scores, but was less effective than MPH [78]. An open-label trial by Uebel-von Sandersleben et al. (2014) reported symptom improvements following the administration of EGb 761^®^, a standardized pharmaceutical-grade extract (the one most used in clinical trials) [79], though the lack of a control group limits conclusions.

Finally, although not conducted in formally diagnosed ADHD patients, a multicenter observational study of 169 primary school children (6–11 years) with attention difficulties, hyperactivity, and impulsivity showed that seven-week treatment with a combination of valerian and lemon balm (Euvegal^®^) led to significant improvements in concentration, restlessness, and behavioral regulation [80].

Although no medicinal plants currently hold an indication for ADHD, several herbal products are traditionally employed to improve attention, concentration, stress response, or mood, domains that are closely related to ADHD symptoms. Indeed, other than the mood- or relaxation-promoting species that have already been presented, the retrieved studies highlight the potential in the management of ADHD symptoms in children of nootropic plants such as *B. monnieri* and *G. biloba*, here primarily considered for its cognitive effects rather than its cerebrovascular properties, as well as the tonic-adaptogen *P. ginseng* and the anxiolytic-adaptogen *W. somnifera*. The literature also highlights some novel perspectives, such as the pharmacological potential of saffron and French maritime pine OPCs.

The risk-of-bias assessment highlighted variability, with RCTs generally rated as “some concerns” due to small sample sizes or incomplete reporting, and open-label or observational studies frequently judged as “high risk”. According to the GRADE framework, the certainty of evidence is overall “low”, indicating the need for further well-designed, adequately powered studies (Table S3). Nevertheless, current data support the hypothesis that phytotherapy may offer a valuable complementary approach in the management of pediatric ADHD.

**Table 3:** Clinical studies using herbal products for ADHD in children. The regulatory status refers to the country in which the study was conducted. RCT: randomized controlled trial.

Study (Author, Year)	Treatment and Regulatory Status	Study Design	Population	Treatment	Main Outcomes
Lee et al. (2021) [48]	Omega-3 + *P. ginseng* extract (Korean red ginseng) granule caps. Herbal material reported in Korean Pharmacopoeia	RCT, double-blind, vs. placebo	120 children (6–12 years) with subclinical ADHD (South Korea)	1 g/day for 12 weeks	Significant improvement in inattention and hyperactivity symptoms in the treatment group compared to placebo
Ko et al. (2014) [49]	Korean red ginseng. Herbal material reported in Korean Pharmacopoeia	RCT, double-blind, vs. placebo	70 children (6–15 years) with ADHD (South Korea)	1 g/day ginseng extract for 8 weeks	Significant reduction in behavioral symptoms compared to placebo
Niederhofer et al. (2009) [50]	Ginseng dry extract (27–30% ginsenosides). Food supplement-grade extract	Open-label clinical trial	3 children with ADHD (Germany)	250 mg extract, orally, once daily for 4 weeks	Improvement in some ADHD symptoms
Dvořáková et al. (2006) [52]	French maritime pine standardized extract (Pycnogenol^®^). Food supplement-grade extract	RCT, double-blind, vs. placebo	43 children (6–14 years) with ADHD (Czech Republic)	1 mg/kg/day for 4 weeks	Increase in plasma glutathione (GSH) levels
Dvořáková et al. (2007) [53]	French maritime pine standardized extract (Pycnogenol^®^). Food supplement-grade extract	RCT, double-blind, vs. placebo	43 children (6–14 years) with ADHD (Czech Republic)	1 mg/kg/day for 4 weeks	Reduction in urinary catecholamine (NA) levels
Trebatická et al. (2006) [54]	French maritime pine standardized extract (Pycnogenol^®^). Food supplement-grade extract	RCT, double-blind, vs. placebo	61 children (6–14 years) with ADHD (Czech Republic)	1 mg/kg/day for 4 weeks	Improvement in inattention and hyperactivity symptoms according to behavioral evaluations
Hosseini et al. (2018) [57]	Withania extract. Not a pharmaceutical-grade extract	RCT, single-blind, vs. placebo	28 children (7–12 years) with ADHD and anxiety symptoms (Iran)	10 mg/day for 6 weeks	Significant reduction in anxiety symptoms and social concerns compared to placebo
Kean et al. (2022) [59]	Bacopa standardized extract (CDRI 08^®^_._) Food supplement-grade extract	RCT, double-blind, vs. placebo	112 children (6–14 years) with ADHD (Australia)	160 mg/day (<35 kg) or 2 × 160 mg (>35 kg) CDRI08^®^ caps for 14 weeks	No significant effects on behavioral symptoms; improvements in mood, cognition, and sleep
Dave et al. (2014) [61]	Bacopa standardized extract (Bacomind^®^). Food supplement-grade extract	Open-label clinical trial	31 children (6–12 years) with ADHD (India)	225 mg/day for 6 months	Significant reduction in scores for ADHD symptoms, except for social problems
Baziar et al. (2019) [70]	Saffron powder. Herbal material used in traditional medicine and as food (not pharmaceutical-grade)	RCT, double-blind pilot study, vs. methylphenidate	54 children (6–17 years) with ADHD (Iran)	20 mg/day (<30 kg) or 30 mg/day (>30 kg) for 6 weeks	Saffron was as effective as methylphenidate in reducing ADHD symptoms, with no significant difference between groups
Khaksarian et al. (2021) [71]	Saffron + methylphenidate. Herbal material used in traditional medicine and as food (not pharmaceutical-grade)	RCT, double-blind, parallel-group, vs. methylphenidate alone	70 children (6–16 years) with ADHD (Iran)	20 mg/day (<30 kg) or 30 mg/day (>30 kg) for 8 weeks	Combination therapy showed greater improvement in ADHD symptoms compared to methylphenidate alone, with significant differences observed after 4 weeks
Blasco-Fontecilla et al. (2022) [72]	Saffron dry extract (>3% crocins and >2% safranal) Saffr’Activ^®^. Food supplement-grade extract	Non-randomized clinical trial, vs. methylphenidate	63 children (7–17 years) with ADHD (Spain)	30 mg/day for 3 months	Saffron was more effective for hyperactivity symptoms, while methylphenidate was more effective for inattention symptoms
Weber et al. (2008) [73]	SJW extract standardized 0.3% hypericin. Food supplement-grade extract	RCT, double-blind, vs. placebo	54 children and adolescents (6–17 years) with ADHD (Germany)	300 mg, orally, three times daily (900 mg/day total) for 8 weeks	No significant improvement in ADHD symptoms compared to placebo
Shakibaei et al. (2015) [77]	Ginkgo standardized extract (Ginko T.D.™) + methylphenidate. Herbal medicine	RCT, double blind, vs. methylphenidate + placebo	66 children and adolescents (7–12 years) with ADHD (Iran)	80–120 mg daily for 6 weeks	Greater reduction in ADHD-RS-IV parent and teacher inattention scores compared to methylphenidate + placebo
Salehi et al. (2010) [78]	Ginkgo standardized extract (Ginko T.D.™). Herbal medicine	RCT, double-blind vs. methylphenidate	50 children (6–14 years) with ADHD (Iran)	80 mg/day for <30 kg and 120 mg/day for >30 kg for 6 weeks	Methylphenidate showed greater improvement in ADHD symptoms compared to ginkgo; fewer side effects observed with ginkgo
Uebel-von Sandersleben et al. (2014) [79]	Ginkgo standardized extract (EGb 761^®^). Herbal medicine	Open-label pilot study	20 children (6–13 years) with ADHD (Germany)	Up to 240 mg daily (titrated over 3–5 weeks)	Improvements in quality of life, in ADHD symptoms, and in Continuous Performance Test
Ross (2015) [80]	Valerian + lemon balm extracts (Euvegal^®^). Herbal medicine	Multicenter, open-label observational study	169 children (4–17 years) with ADHD (Germany)	640 mg valerian + 320 mg lemon balm, orally, twice daily for 7 weeks	Significant change in concentration scores, hyperactivity, and impulsiveness

#### 3.2.4. Herbal Products for Depressive Symptoms

Major depressive disorder (MDD) in youth is a significant public health issue, with early onset linked to long-term emotional, cognitive, and social impairments. While pharmacological and psychotherapeutic treatments remain first-line options, there is growing interest in complementary approaches, particularly medicinal plants, for mild to moderate forms or when standard therapies are unsuitable. Within this context, two clinical trials were identified in our analysis: one focused on pediatric MDD and another on mixed anxiety-depressive symptoms (Table 4).

A RCT versus placebo evaluated the efficacy of affron^®^, a standardized food supplement-grade saffron extract, in adolescents aged 12–16, with outcomes measured by the Revised Child Anxiety and Depression Scale (RCADS). After eight weeks, the treatment was well tolerated and significantly improved anxiety and depressive symptoms in youth, based on their self-reports. These beneficial effects were, however, inconsistently corroborated by parents [81].

A second open-label pilot study investigated the use of an SJW extract, marketed in the USA as a dietary supplement, in children and adolescents with MDD [82]. After eight weeks, 25 out of 33 participants showed a clinical response on the Children’s Depression Rating Scale-Revised (CDRS-R) and the Clinical Global Impressions (CGI), although 22 children required dose escalation after 4 weeks. The treatment was well tolerated.

Taken together, the evidence regarding medicinal plants for pediatric MDD is highly preliminary. Overall, the risk-of-bias assessment ranges from “some concerns” to “high risk”, and, according to the GRADE approach, the certainty of evidence is judged “very low” (Table S3).

These findings underscore the need for larger, well-designed trials before any clinical recommendation can be made.

#### 3.2.5. Herbal Products for Autism Spectrum Disorder and Dyslexia

Autism spectrum disorder (ASD) is a complex neurodevelopmental condition marked by deficits in social interaction, communication, and behavior. Common symptoms such as irritability, hyperactivity, and agitation can greatly affect quality of life. Growing interest in natural remedies highlights their potential as adjuncts to conventional therapies in the management of symptoms (Table 5). A randomized, double-blind, RCT versus placebo by Hasanzadeh et al. (2012) evaluated the addition of the pharmaceutical-grade dry *G. biloba* extract Ginko™ to risperidone in children with ASD [83]. Outcomes measured by the Aberrant Behavior Checklist-Community (ABC-C) rating scale were similar in all five subscales for the two groups (ginkgo + risperidone and placebo + risperidone), suggesting that ginkgo did not affect the conventional treatment efficacy.

Individuals with ASD may also have comorbid dyslexia, a specific learning disorder characterized by difficulties with reading, spelling, and phonological processing. While traditional interventions focus on educational strategies, complementary approaches are being explored. A pilot study by Donfrancesco et al. (2007) investigated the effects of *G. biloba* extract EGb761^®^ in children with dyslexia [84]. After the eight-week trial, the children showed significant improvement on standardized tests for reading words and non-words and reading text, with half of them no longer meeting the diagnostic criteria for dyslexia. 

Although the number of studies is limited, the existing evidence primarily focuses on *G. biloba*, recognized for its general neuroprotective properties, suggesting potential avenues for adjunctive support in children with ASD and related learning difficulties.

Studies including both ASD and dyslexic populations are few and heterogeneous. The RCT on ASD by Hasanzadeh et al. presents “some concerns” due to the small sample size and incomplete reporting, while the pilot study on dyslexia is “high risk” due to its uncontrolled, open-label design. According to the GRADE framework, the overall certainty of evidence is “very low” to “low”, reflecting the need for further well-designed, adequately powered trials, yet suggesting preliminary potential for *G. biloba* as a neuroprotective adjunct in pediatric neurodevelopmental conditions (Table S3).

### 3.3. Herbal Products Emerged from Clinical Studies

Several medicinal plants emerged from the clinical studies reviewed, with particular interest in the following species: *Bacopa monnieri* (L.) Wettst., *Crocus sativus* L. stigma, *Ginkgo biloba* L. folium, *Hypericum perforatum* L. herba, *Lavandula angustifolia* Mill. aetheroleum, *Melissa officinalis* L. folium, *Panax ginseng* C.A. Meyer radix, *Passiflora incarnata* L. herba, *Pinus pinaster* Aiton. Cortex, *Valeriana officinalis* L. radix, and *Withania somnifera* (L.) Dunal radix. As mentioned before, while most preparations were marketed as food supplements, the standardized extracts of *H. perforatum* and *G. biloba* and the valerian-lemon balm combination are approved as medicinal products and included in EMA monographs.

The next section examines the pharmacological activities of selected botanicals with relevance to agitation, restlessness, hyperactivity, and mood disturbances, commonly encountered in various neuropsychiatric conditions in youth, based on both published data and predictive molecular target analyses (Table S4).

#### 3.3.1. *Bacopa monnieri* (L.) Wettst Herba

Commonly known as brahmi, this is a creeping herb (Plantaginaceae) traditionally used in Ayurvedic medicine to enhance memory, learning, and cognitive performance [85]. Although not included in the European Pharmacopoeia, bacopa is included in the Indian pharmacopoeia and in the United States Pharmacopoeia-National Formulary (USP-NF) (the herbal substance should not contain less than 2.5% of total tritepenes glycosides as the sum of bacopaside I, bacoside A_3_, bacopaside II, the jujubogenin isomer of bacopasaponin C, and bacopasaponin C.). In the EU, bacopa is authorized as a food supplement for neurocognitive support, typically in the form of standardized extracts with a quantified content of bacosides (collectively referred to as bacopa triterpene glycosides).

The phytochemical composition of *B. monnieri* includes a broad range of bioactive compounds, including bacosides, saponins, alkaloids, flavonoids, and sterols [86]. These constituents exert antioxidant, anti-inflammatory, and anti-apoptotic effects, contributing to neuroprotective activity [87]. In particular, bacosides enhance mitochondrial function and modulate key neurotransmitter systems, including serotonergic and glutamatergic pathways, notably in the hippocampus [88]. Bioinformatic analyses showed that main constituents of bacopa, such as bacopaside I, bacopaside II, and bacoside A3, have a low ability to cross the blood–brain barrier (BBB), which is slightly better for aglycones such as jujubogenin and bacogenin A1 derived from bacosides after gastrointestinal digestion.

GeneCards analysis revealed that the main constituents of *B. monnieri*, including aglycones and glycosylated compounds, mainly converged in targeting BDNF-antisense (BDNF-AS). Bioinformatic tools operating through molecular similarities and chemical properties (STP and SEA) showed that bacopa constituents did not produce a high score of probability in specifically targeting genes or protein. Instead, SuperPred revealed some common targets plausibly associated with the main constituents of *B. monnieri* related to stress, anxiety, mood disorders, or cognitive functions or neuroactivity. Specifically, a plausible role of the bacopa phytocomplex emerged in modulating multiple targets mainly related to neuroprotection and cognitive function, including the neurotrophic factor neurotrophin 3 (NT-3), epigenetic regulators (histone deacetylase 1 and 2 (HDAC1, HDAC2)), neurotransmitter metabolism (voltage-gated N-type calcium channel alpha-1B subunit (CaV2.2 1B) and G-protein coupled receptor 55 (GPR55)), and immune modulation (e.g., nuclear factor NF-kB p105 subunit (NF-κB p105), cannabinoid receptors 1 and 2 (CB1r/CB2r)) (Table S4). Interaction with glutamatergic receptors was not confirmed in silico. The data suggest a broad modulatory effect on pathways relevant to cognitive function, stress response, and neuroinflammation (Table S4).

Together, these findings highlight the complex and pleiotropic pharmacological profile of *B. monnieri*, supporting its use as a neuroprotective agent with potential applications in cognitive and mood disorders.

#### 3.3.2. *Crocus sativus* L. Stigmata

*Crocus sativus* L. (saffron), a perennial monocot of the Iridaceae family, is primarily valued for its dried stigmas, which are rich in the carotenoid-derived and terpenoid compounds responsible for its color, taste, and aroma. The main constituents include crocins, water-soluble diesters of crocetin and gentiobiose, which contribute to saffron’s characteristic yellow hue and account for 6–16% of its dry weight. Crocetin, the aglycone of crocins, exhibits antioxidant activity. Picrocrocin imparts bitterness, while safranal, formed during drying, is the main aromatic component. Minor lipophilic carotenoids such as lycopene, α-/β-carotene, and zeaxanthin are also present.

Although no saffron-based drugs are currently authorized in the EU, *C. sativus* has a long tradition of use across the Middle East, India, and parts of Europe for gastrointestinal, neurological, and ocular conditions [89,90]; It can be used also in food supplements.

Modern pharmacological studies have identified several mechanisms underlying its neuroactive effects: (i) inhibition of serotonin and norepinephrine reuptake (mainly by crocins and safranal), (ii) modulation of GABA-A receptors, (iii) antagonism of glutamatergic signaling, (iv) hypothalamic-pituitary-adrenal (HPA) axis regulation, and (v) pronounced antioxidant and anti-inflammatory activity [91,92]. Both crocins (especially in the aglycone form of crocetin formed after oral ingestion) and safranal have been reported to cross the BBB in experimental models and reach the systemic circulation.

In silico analyses suggested interactions of crocins and safranal with targets such as TNF-α, catalase, and BDNF-AS, supporting roles in oxidative stress modulation and neuroplasticity (Table S4). However, no high-affinity single molecular targets were identified, reflecting the complex and multitarget pharmacodynamic profile of saffron.

#### 3.3.3. *Ginkgo biloba* L. Folium

*Ginkgo biloba* L., the only extant species of the Ginkgoaceae family, is a long-lived gymnosperm with a well-established history of medicinal use in Asia and, over the past 50 years, in Western countries. The part used medicinally is the dried leaf, which is rich in a distinctive phytocomplex composed of diterpenes (notably ginkgolides A, B, C, J, and M), sesquiterpene lactones (bilobalide), proanthocyanidins, and flavonoid glycosides (mainly quercetin and kaempferol derivatives), with ginkgolic acids present only in traces in pharmaceutical-grade extracts. According to the European Pharmacopoeia, standardized *G. biloba* extracts typically contain 6–7% terpenes and 24% flavonoids, and are authorized in the EU for improving age-associated cognitive impairment and quality of life in patients with mild dementia [93]. Other standardized dry extracts with similar chemical characteristics are marketed as herbal medicines in other countries in Asia. Preparations from this species are also approved for use as food supplements in the EU, the USA, and many countries worldwide. The pharmacological activity of *G. biloba* is multitarget, involving antioxidant, anti-inflammatory, and neuroprotective mechanisms [94,95]. Flavonoids counteract reactive oxygen species (ROS) and lipid peroxidation, while ginkgolides and bilobalide protect mitochondrial function and inhibit caspase activation. Additionally, the extract promotes vasodilation and modulates cholinergic, dopaminergic, and serotonergic pathways, supporting cognitive and emotional regulation [74,96].

From the literature, and by refining the search by means of SwissADME, we found that, among the active components included in the ginkgo phytocomplex, bilobalide was predicted to be absorbed in the gastrointestinal tract and to cross the BBB; also, ginkgolides could be partially distributed to the brain, as well as flavonol aglycones derived from native glycosides. GeneCards revealed several converging targets among the main constituents, including mitogen-activated protein kinase 2 (ERK2), CAMP responsive element binding protein 1 (CREB1), CC motif chemokine ligand 2 (CCL2), aryl hydrocarbon receptor (AHR), 5-hydroxytryptamine receptor 3A (5-HT3A), and synuclein-α (Table S4). STP and SEA showed two distinctive patterns of targets related to flavonoids and terpenes involved in brain functions: terpenes were predicted to target glycine receptor subunits 1 and 2 (GLR1A/GLR2A) and, with lower scores, platelet-activating factor receptor (PAFR), while flavonols were linked to the vascular endothelial growth factor (VEGFR), monoamine oxidase A (MAO-A), A1/A2A adenosine receptors (A1AR and A2AR), dopamine receptor D4 (D4R), protein kinase B (AKT), and phosphoinositide-3-kinase (PI3K) pathways, as well as inflammatory mediators (cyclooxygenase (COX), lipoxygenase (LOX), IL-8, and matrix metalloproteinases (MMPs)) (Table S4). SuperPred further confirmed MAO-A and 12-lipoxygenase (12-LOX) as flavonol targets and identified neuropeptide 5 receptor (NP5R) and endoplasmic reticulum-associated amyloid b-peptide-binding protein (ERAB) as specific targets of quercetin. As regards ginkgolides (focused on ginkgolide B) and bilobalide, the converging targets were platelet-derived growth factor receptor A (PDGFRA), hypoxia inducing factor 1 (HIF-1), CB2R, and Cav2.2 1B (Table S4).

These findings support the complex pharmacodynamics of *G. biloba*, with overlapping actions affecting oxidative stress, neuroinflammation, neurotransmission, and growth factor signaling. The predicted targets align with known neuroprotective effects and suggest a possible role in the modulation of mood and agitation symptoms.

#### 3.3.4. *Hypericum perforatum* L. Herba

*Hypericum perforatum* L. (SJW) is a perennial herb of the Hypericaceae family, traditionally used to treat mild to moderate depression, nervous tension, and sleep disturbances [97,98,99]. It is one of the most thoroughly investigated medicinal plants in Europe and is included in the European Pharmacopoeia. Standardized extracts are characterized by defined levels of hypericins (0.10–0.30%), flavonoids (≥6.0%, expressed as rutin), and hyperforin (max. 6.0%). SJW extracts are marketed as conventional or well-established medicines in the EU, indicated for mild to moderate depressive symptoms [100], though in the pediatric populations they are more often found in food supplement formulations. SJW contains a complex phytochemical profile with CNS-relevant activity: its main bioactive constituents include phloroglucinols (hyperforin, adhyperforin), naphthodianthrones (hypericin), flavonoids (biflavones such as amentoflavone and flavonols such as hyperoside and rutin), proanthocyanidins, and phenolic acids.

Our pharmacokinetic predictions, in line with the existing literature, indicate that the native constituents of SJW show limited ability to cross the BBB. However, several metabolites appear capable of BBB penetration, including flavonol aglycones, proanthocyanidin-derived metabolites such as (–)-epicatechin and (+)-catechin metabolites, 5-(3′,4′-dihydroxyphenyl)-γ-valerolactone, and small hydroxycinnamic acids such as caffeic and ferulic acid (Table S4).

According to GeneCards, hyperforin (and adhyperforin), hypericin, and quercetin (the aglycone form of hyperoside and rutin) were all associated with ERK1/2, VEGFA, BDNF-AS, IL-8, and IL-6. Notably, similar predictions were observed for (–)-epicatechin, (+)-catechin, and caffeic acid. Amentoflavone also targeted ERK2 (Table S4). Although the STP and SEA tools provided few high-confidence predictions, with the exception of flavonols, already discussed for *G. biloba*, SuperPred suggested that most SJW compounds, excluding hyperforin but including (–)-epicatechin, 5-(3′,4′-dihydroxyphenyl)-g-valerolactone, and caffeic and ferulic acid—may target MAO-A and, with weaker scores, GLRA1 and VEGFA. Instead, hyperforin was predicted to modulate NF-kB p105, ERK2, CB2R, GPR55, and, with moderate confidence, sodium channel proteins (Table S4). These predictions, combined with literature evidence, support a multitarget mechanism involving modulation of neuroinflammation and neurotransmitter systems, notably reuptake inhibition and signal transduction regulation.

#### 3.3.5. *Lavandula angustifolia* Mill. Aetheroleum

*Lavandula angustifolia* Mill. (“true lavender”), an aromatic plant from the Lamiaceae family, is widely used for its anxiolytic and sedative properties. Its main preparation is lavender essential oil (lavender oil), extracted by steam distillation of the flowering tops. According to the European Pharmacopoeia, lavender oil must contain at least 20% linalool and 25% linalyl acetate, the monoterpenes primarily responsible for its fragrance and therapeutic effects, along with terpinen-4-ol as a third major constituent [101].

The pharmacological effects of lavender oil depend on the route of administration. Inhalation, bath use, or transdermal application can induce rapid anxiolytic effects in cases of mild or transient symptoms, while oral administration ensures more sustained therapeutic activity [102]. In line with this, EMA monographs report the availability of traditional herbal medicinal products containing oral lavender oil (e.g., soft capsules) indicated for mild stress, exhaustion, and sleep disturbances [103]. Lavender oil is available also in food supplements and, for inhalation, as fragrance.

Mechanistically, lavender oil constituents act on multiple CNS targets. Linalool and linalyl acetate positively modulate GABA-A receptors and antagonize NMDA-type glutamate receptors, reducing excitatory transmission and promoting neuroprotection and mood regulation. Additionally, linalool inhibits the serotonin transporter (SERT), enhancing serotonergic signaling [104,105]. Inhalation also engages the olfactory-limbic pathway, influencing dopamine, serotonin, and GABA release and contributing to the rapid onset of anxiolytic effects [106,107,108].

Bioinformatic analyses confirmed the CNS bioavailability of lavender terpenes. GeneCards identified common targets for lavender terpenes such as the transient receptor potential melastatin 8 (TRPM8) and the glutamatergic NMDA receptor subunit 2B. SEA, STP, and Super Pred, however, did not highlight common targets with high or medium prediction scores, supporting a multimodal mechanism involving neurotransmitter modulation and neuroprotection (Table S4).

#### 3.3.6. *Melissa officinalis* L. Folium

*Melissa officinalis* L. (lemon balm), a perennial herb from the Lamiaceae family, is widely used in the Mediterranean and temperate regions. Its medicinal use is well documented both in traditional practice and in regulatory references such as the European Pharmacopoeia (dried leaves should contain not less than 1.0% of rosmarinic acid, according to the monograph issued in the 11th ed.) and EMA monographs have reported the use of different preparations, such as the dry extract (not less than 2.0% of rosmarinic acid, according to the European Pharmacopoeia 11th ed.) [93,109]. In the EU, lemon balm preparations, particularly in combination with valerian (e.g., Euvegal^®^), are authorized for relieving mild stress and aiding sleep, as well as for gastrointestinal complaints [110,111,112]. Lemon balm is also commonly available in herbal teas and in food supplements in different preparations.

The leaves are rich in polyphenols, including hydroxycinnamic acids (rosmarinic, chlorogenic, caffeic), flavonoids (apigenin, luteolin, quercetin glycosides), triterpenes (ursolic, oleanolic acids), and volatile compounds (citral, neral, citronellal). In dry extracts, rosmarinic acid is the main active constituent, while the essential oil content is negligible. The phytocomplex exerts CNS activity mainly via GABAergic and cholinergic modulation. Rosmarinic acid inhibits GABA transaminase and may bind GABA-A receptors, although the relevance of this effect, in vivo, remains debated [113]. Additionally, gut–brain axis involvement is increasingly recognized among its effects [114].

SwissADME analysis and the literature highlighted that lemon balm compounds have low oral bioavailability, and only rosmarinic acid metabolites like caffeic and ferulic acid, as well as some flavonoids as aglycones, likely reach the CNS to produce pharmacological effects [115]. GeneCards identified BDNF-AS as a shared CNS target of caffeic acid derivatives and flavonoids. For luteolin and apigenin, additional predicted targets included nuclear factor erythroid 2-related factor 2 (Nrf2), NF-κB subunit 1, ERK2, signal transducer and activator of transcription 3 (STAT3), HIF-1α, caspase-8, and caspase-9, all of which are involved in neuroinflammation and neuronal survival. The STP and SEA tools identified MMP-1, MMP-9, and 5-LOX as targets of hydroxycinnamic acids, with high predictivity scores. Flavonoids (apigenin, luteolin, quercetin) were also predicted to target MMP-9, MMP-12, cyclin-dependent kinase 5 (CDK5) activator, MAO-A, A1/A2 adenosine receptors, 12-/15-LOX, acetylcholinesterase, and COX-2 (Table S4). The convergence in targeting MMP-9 suggested a role for the lemon balm phytocomplex in extracellular matrix remodeling and immune regulation.

SuperPred further confirmed MAO-A as a central mood-related target for both hydrocinnamic acids and flavonoids, along with Nrf2, NF-κB p105, ERK2, and glycine transporter 2 (GlyT2) for hydroxycinnamic acids and GLR1 and 12-LOX for flavonoids (Table S4).

Collectively, these findings highlight *M. officinalis* as a multitarget phytocomplex with neuroprotective, anti-inflammatory, and mood-modulating potential, partly mediated by MAO-A inhibition and metalloprotease regulation, as previously proposed by López et al. (2009) [116].

#### 3.3.7. *Panax ginseng* C.A. Meyer Radix

*Panax ginseng* C.A. Meyer (Asian ginseng), a perennial plant of the Araliaceae family, has been used for centuries in traditional Asian medicine and is recognized today as one of the most important tonic-adaptogen herbs. Native to northeastern Asia, it is prepared as white ginseng (oven-dried) or red ginseng (steam-treated); the latter is commonly used in Korea. The EMA supports its traditional use for temporary fatigue and weakness, and both ginseng root and dry extract are included in the European Pharmacopoeia (11th ed.), with specifications based on ginsenoside content (≥0.4% in raw material, ≥4% in dry extract) [93,117]. Formulations based on ginseng roots are also marketed as ingredients for food supplements, alone or in combination with other adaptogens or nootropics.

Ginseng’s pharmacological activity is attributed primarily to two compound classes: ginsenosides, with CNS activity [118,119], and polysaccharides, known for immunomodulatory and prebiotic properties [120]. Its mechanism of action is pleiotropic and multitarget, consistent with its adaptogenic nature. Key mechanisms include modulation of the HPA axis [121], regulation of oxidative stress and mitochondrial function in neurons [122], and peripheral effects on immune function and glucose metabolism [120].

Ginsenosides are metabolized in the gut and liver into active triterpenoid derivatives such as protopanaxadiol (PPD), protopanaxatriol (PPT), compound K, and the ginsenosides Rg3, Rf1, Rh1, which, according to our pharmacokinetic predictions and the existing literature, may reach the CNS [123].

Bioinformatic analyses revealed several shared CNS-related targets. GeneCards identified BDNF-AS, AKT1/2/3, mTOR, and NR3C1 (glucocorticoid receptor) as key regulatory nodes. While STP and SEA did not yield high-confidence converging targets, SuperPred predicted interactions with PKC, butyrylcholinesterase (BCHE), A1AR, HDAC2, vitamin D receptor (VDR), NT-3, and components of the NF-κB pathway, particularly NF-κB p105 and mineralocorticoid receptor (MR) (Table S4).

Additional targets included proteins involved in neuronal excitability and neuroimmune signaling, such as SCN2A (associated with sodium channels), CB2, GPR55, and, to a lesser extent, CB1, although these require further validation (Table S4).

Overall, these data support a multimodal neuroprotective mechanism of ginsenosides, with convergence on pathways regulating neurotrophism, inflammation, neuroplasticity, and stress resilience.

#### 3.3.8. *Passiflora incarnata* L. Herba

*Passiflora incarnata* L. (passionflower), a perennial climber of the Passifloraceae family, is native to the Americas and widely distributed across temperate and subtropical regions. It has a long tradition of medicinal use and is officially included in the European Pharmacopoeia (11th ed.), with a monograph on its aerial parts (containing not less than 1.5% total flavonoids expressed as vitexin) and other related herbal preparations such as the dry extract, standardized to contain ≥2.0% flavonoids in the herb (expressed as vitexin) [93,124]. In the EU, *P. incarnata* preparations are marketed both as traditional herbal medicinal products for mild mental stress and sleep disturbances and as a botanical supplement.

The pharmacologically active compounds are mainly C-glycosyl flavonoids, including isovitexin, isoorientin, shaftoside, vitexin, and orientin, alongside minor constituents such as β-carboline alkaloids (e.g., harman), cyanogenic compounds, and volatile terpenes [125,126,127,128,129].

The main mechanism of action involves positive modulation of GABA-A receptors, likely mediated by C-glycosyl flavonoids, which also enhance glutamate decarboxylase (GAD) activity and increase GABA levels [130,131]. Preclinical studies have confirmed GABAergic effects in key brain regions such as the hippocampus and hypothalamus. Some constituents, including β-carboline alkaloids, may also modulate opioid receptors, as indicated by naloxone-sensitive analgesia [132]. Additionally, spasmolytic effects on smooth muscle may underlie its benefit in gut-related nervous symptoms.

SwissADME analysis suggests limited BBB permeability for C-glycosides, whereas aglycones (e.g., luteolin, apigenin) and their metabolites likely mediate CNS effects. GeneCards identified >150 targets shared by apigenin and luteolin, including caspase 8 and 9 (CASP8, CASP9), STAT3, Nrf2, NF-κB subunit 1, HIF-1α, and BDNF-AS, all of which are implicated in neuroprotection and neuroinflammation (Table S4).

The STP and SEA tools confirmed targets relevant to mood and cognitive function, such as MAO-A, A1AR, A2AR, acetylcholinesterase, MMP-9, MMP-12, CDK5 activator, and inflammatory enzymes (5-, 12-, 15-LOX, COX-2). SuperPred further supported MAO-A, 12-LOX, GLR1, AMPA receptor 2 (AMPA-2), and sodium- and chloride-dependent glycine transporter 2 (GlyT2) as targets with medium to high probability for both aglycones and their glycosides (Table S4).

A direct interaction with GABA-A receptors was not predicted by in silico tools, although this mechanism is experimentally supported, suggesting that *P. incarnata* acts through a complex, multitarget mode involving GABAergic modulation, monoamine regulation (MAO-A), inflammatory and oxidative stress pathways (Nrf2, NF-κB, LOX), and extracellular matrix remodeling (MMPs).

#### 3.3.9. *Pinus pinaster* Aiton Cortex

*Pinus pinaster* Aiton (French maritime pine) (Pinaceae) is a coniferous species native to the western Mediterranean. Dry bark extracts, with Pycnogenol^®^ being the most studied, are rich in OPCs, catechins, phenolic acids, and cinnamic acid derivatives. Though widely marketed in Europe as an ingredient in food supplements for cognitive support, microcirculation, and skin health [133,134,135], Pycnogenol^®^ or other preparations of *P. pinaster* are not currently included in the European Pharmacopoeia or authorized as a traditional herbal medicinal product by the EMA.

The extracts, which are enriched in OPCs, exert antioxidant, anti-inflammatory, and vasoprotective effects, though the mechanism of action is not fully elucidated [136,137,138]. Human studies have identified some main circulating metabolites found in plasma after oral administration of Pycnogenol^®^: (+)-catechin, caffeic acid, ferulic acid, and 5-(3′,4′-dihydroxyphenyl)-γ-valerolactone. These compounds are also present in *H. perforatum* and *M. officinalis*. These metabolites show limited but plausible CNS penetration and are believed to contribute to the neuroprotective and cognitive effects of *P. pinaster* extracts [135,136,139].

Bioinformatic analysis revealed overlapping CNS targets for (+)-catechin, caffeic acid, and ferulic acid (not shared by 5-(3′,4′-dihydroxyphenyl)-γ-valerolactone). GeneCards identified shared targets, including BDNF-AS, catalase, MMP-9, ERK2, Nrf2, iNOS, EGFR, and HIF-1α. STP and SEA predicted MMP-1, MMP-9, and 5-LOX as relevant targets for hydroxycinnamic derivatives, whereas no high-score predictions were identified for 5-(3′,4′-dihydroxyphenyl)-γ-valerolactone or (+)-catechin (Table S4). SuperPred confirmed MAO-A as a recurring high-scoring target and also suggested NF-κB p105 and GLRA2 as additional targets. Overall, these data support a multitarget mechanism involving oxidative stress modulation, neuroinflammation, and MMP activity. In particular, MMP-9 and MAO-A emerged as key targets, aligning with the observed effects on synaptic plasticity and neurovascular integrity [135,140].

#### 3.3.10. *Valeriana officinalis* L. Radix

*Valeriana officinalis* L. (valerian) is a perennial herb of the Caprifoliaceae family, and is historically and clinically well-documented in Europe for the treatment of mild anxiety, nervous tension, and sleep disorders [141,142,143]. Its pharmacological activity is primarily attributed to the phytocomplex extracted from the underground organs (*Valerianae radix*), officially recognized by the European Pharmacopoeia (11th ed.), which sets quantitative standards for sesquiterpenic acids (e.g., valerenic acid): ≥0.17% in the root, ≥0.25% in ethanolic dry extracts, and ≥0.02% in aqueous extracts [93].

The root contains sesquiterpenes (valerenic, hydroxyvalerenic, acetoxyvalerenic acids), iridoids (e.g., valepotriates and their degradation products baldrinal and valeric acids), lignans, volatile monoterpenes, organic acids, free GABA, and polysaccharides. In the EU, valerian is used both in registered medicines (often combined with *M. officinalis* or *H. lupulus*) and in food supplements [144].

Valerenic acid is considered the principal bioactive compound, acting as a positive allosteric modulator of GABA-A receptors at a non-benzodiazepine binding site, promoting anxiolytic and sedative effects without tolerance or dependence. It also enhances GABA synthesis via glutamate decarboxylase (GAD) activation. Additional interactions have been suggested with A1AR and serotonergic pathways, although hydroxy- and acetoxy-valerenic acids may attenuate GABAergic effects, highlighting the importance of extract standardization [112].

Pharmacokinetic modelling indicates that valerenic acid and its minor derivatives, along with the baldrinal, valeric, and isovaleric acids, can cross the BBB. However, due to their low and variable concentrations, GABA and lignans are unlikely to significantly contribute to its central effects. Therefore, computational predictions were focused on valerenic acids.

GeneCards analysis identified GABA-A1, GABA-A2, GABA-B1, and 5-HT-5A as primary targets of valerenic acid. While STP and SEA did not yield high-confidence predictions, SuperPred suggested possible interactions with NF-κB p105 and, to a lesser extent, NMDA receptors (Table S4). These findings support multifaceted yet modest CNS activity, with no single dominant mechanism, consistent with the relatively low active compound content in most valerian-based formulations.

#### 3.3.11. *Withania somnifera* (L.) Dunal Radix

*Withania somnifera* (L.) Dunal, commonly known as ashwagandha or Indian ginseng, is a perennial shrub of the Solanaceae family, traditionally used in Ayurvedic and Unani medicine as a rasayana, a tonic to promote longevity, vitality, cognitive enhancement, and stress resilience [145,146,147]. The dried root (*Withaniae radix*) is the principal medicinal part. While the WHO recognizes its use for relieving symptoms of stress, fatigue, and insomnia [148], *W. somnifera* is not authorized as a medicinal product by the EMA, but it is officially included in some Asian pharmacopoeias (as in India) and in the USP-NF (dry extract should contain not less than 2.5% total withanolides calculated as sum of aglycones and glycosides), and it is authorized within the EU for the formulation of food supplements.

Recently, high-quality standardized extracts (with ≥2.5–5% withanolides) have become widely available and are the basis for most clinical studies.

Pharmacologically, *W. somnifera* contains a diverse phytochemical profile, notably withanolides (e.g., withaferin A, withanolide A, withanone), withanosides, and alkaloids (e.g., isopellieterine, anaferine). These constituents contribute to its adaptogenic, neuroprotective, anti-inflammatory, and anxiolytic properties. Preclinical and clinical studies have demonstrated the ability of withania to modulate the HPA axis, reduce cortisol, and enhance antioxidant defenses in neural tissue [149,150,151]. Withanolides also influence GABAergic and cholinergic neurotransmission and support neurotrophic signaling, making the extract relevant for stress, anxiety, and cognitive impairment [152,153].

Pharmacokinetic data suggest that withanolides, withaferin A, withanone, and alkaloids can partly cross the BBB, whereas withanosides do not reach the CNS [154,155].

GeneCards analysis did not provide valid results, as only a match between the term “withanolides” and withaferin A could be made; however, this intersection showed that the common targets related to the CNS with high scores included AKT1, CASP3, heat-shock protein 90 (HSP90), NF-κB subunit 1, IκB kinases (IkBK)β and γ, BDNF-AS, SOD-2 overlapping transcript 1 (SOD2-OT1), and IL-6 (Table S4).

Although SEA did not provide reliable predictions, STP showed distinct targets for withanolides/withaferin A and derivatives and alkaloids, with withania triterpenes having several predicted targets, including IκBK, PKC, and COX-2, but with low scores, and alkaloids mainly related to neuronal acetylcholine receptors (CHRNA), with low/medium scores (Table S4).

SuperPred predictions revealed overlapping targets for both terpenes and alkaloids, notably NF-κB p105 and CB2R. For withanolides, additional targets included CaV2.2 (1B), NT-3, HDAC-10, HDAC-2, and androgen receptor (AR). Alkaloids were predicted to act on SCN2A and on ADRB1 (Table S4).

Altogether, *W. somnifera* exhibits a multitarget pharmacological profile with strong convergence on neuroinflammatory and neuroprotective pathways, consistent with preclinical findings [153]. This supports its classification among adaptogenic and nootropic botanicals and suggests its potential in managing stress-related and cognitive disorders, even in the pediatric populations.

## 4. Discussion

The mental well-being of children is a critical domain that must be addressed proactively rather than passively. While restoring balance in cases of emotional or behavioral dysregulation is essential, it is equally important to avoid overmedicalization, particularly when symptoms are mild, fluctuating, or subthreshold [156,157]. Conditions such as subthreshold anxiety, agitation, sleep disturbances, or early attentional dysregulation rarely justify immediate pharmacotherapy. In these contexts, conventional psychotropic drugs may be disproportionate, carrying risks of adverse events, dependence, and stigmatization. Medicinal plants, when used rationally, offer a valuable middle ground between clinical inertia and pharmaceutical escalation [158,159]. EMA recognizes several herbal medicines for mild anxiety, stress, and sleep disorders, and many botanical supplements are authorized for non-pathological pediatric use [34]. Despite this regulatory recognition, the scientific evidence base remains limited and heterogeneous in children and adolescents. This is largely due to ethical constraints, developmental variability, and methodological challenges in pediatric research. This review aimed to fill that gap by systematically appraising clinical trials of herbal products in children and adolescents, distinguishing between formal psychiatric diagnoses and subthreshold or functional symptoms. Although fragmented, the evidence is informative (Table 6).

Notably, ADHD is the best-studied pediatric condition for the use of herbal products, likely due to the considerable side effects associated with conventional pharmacotherapy such as methylphenidate or atomoxetine [7].

Although evidence still needs to be strengthened, phytotherapy has emerged as a pragmatic option, particularly in early or mild presentation of symptoms, demonstrating potential to delay, reduce, or complement pharmacological treatments, with generally favorable tolerability.

The analysis of studies of ADHD provides an opportunity to examine medicinal plants with distinct pharmacological profiles, thereby also highlighting potential connections with other disorders. Tonic-adaptogens such as *P. ginseng*; anxiolytic-adaptogens like *W. somnifera*; nootropic and antioxidant agents like *B. monnieri*, *P. pinaster*, and *G. biloba*; classical anxiolytics and sedatives such as *V. officinalis* and *M. officinalis*; and antidepressants, namely *H. perforatum* and *C. sativus*, were evaluated in children with ADHD.

The role of adaptogens in mental stress and weakness is well-established and taken into account in clinical trials found in this study. *P. ginseng* is the adaptogen mainly considered in clinical trials found in this study, evaluated both in Asia and in Europe: this did not represent a surprise, considering that this medicinal plant is one of those with the longest tradition of use in TCM, it is officially recognized in several official Pharmacopoeias, it is also present in herbal medicines authorized by the EMA in the EU, and it is very exploited in the sector of food supplements worldwide. Preliminary results provide positive perspectives for its use in children with symptoms related to ADHD. However, the use of *P. ginseng* in children warrants special caution due to metabolic and endocrine effects, as reminded by Ethe MA in its assessment and EU monograph [119]. Another adaptogen has been evaluated in children with symptoms related to ADHD: *W. somnifera*, a key medicinal plant traditionally used in Ayurveda. To date, only one RCT has evaluated this medicinal plant in children, and its use is almost exclusively limited to food supplements. However, a growing body of adult studies supports the efficacy and safety of standardized extracts of *W. somnifera* (such as KSM-66^®^, the most studied) in the management of stress and anxiety [152,153,155]. These findings suggest the need for further investigations in pediatric populations.

Tradition and well-established use are particularly relevant for nootropic plants aimed at supporting cognitive functions. Among them, standardized *G. biloba* extracts—widely authorized as herbal medicines for adults in EU and non-EU countries—have also been investigated in pediatric populations, both alone and in combination with conventional therapies, for disorders such as ADHD, autism, and dyslexia. Measured outcomes provided heterogenous results and inconclusive evidence of the efficacy of *G. biloba* in relieving neuro-psychiatric symptoms in children [77,78,79,84], despite its demonstrated pharmacological potential [76,94,160]. Although in the clinical trials considered in this study *G. biloba* extracts were generally well tolerated, as with *P. ginseng*, and even more so with *G. biloba*, caution is warranted in pediatric use due to the potential risks of drug interactions and bleeding [161,162]; indeed, from a regulatory perspective, ginkgo-containing medicinal products in the EU are only authorized from the age of 18. *G. biloba* was not the only neuroprotective and nootropic plant taken into consideration in clinical trials in children with ADHD; in fact, *B. monnieri* and the *P. pinaster* standardized extract Pycnogenol^®^ were also evaluated, with the latter providing some preliminary positive results in small RCTs in which psychological and biological parameters were evaluated [52,53,54].

In children with ADHD, plants with anxiolytic and sedative properties have been investigated to address symptoms such as agitation and insomnia. The EU-authorized herbal medicine Euvegal^®^ (a fixed combination of standardized extracts of *V. officinalis* and *M. officinalis*) has been studied in children with subthreshold ADHD symptoms, restlessness, and sleep disturbances, involving the largest pediatric samples and showing encouraging results, although evidence remains very limited due to observational designs without placebo controls [38,39,80,112]. A non–pharmaceutical-grade combination of *V. officinalis, P. incarnata,* and *H. perforatum* has also been investigated for nervous agitation. From a pharmacological perspective, combinations of *V. officinalis* with *M. officinalis* or *P. incarnata* are of particular interest, as these plants are well tolerated and exert both central calming and gastrointestinal spasmolytic effects, potentially enhancing tension relief [115,163].

*H. perforatum* remains the best-characterized herbal antidepressant, but its use remains controversial, especially in children: in fact, medicines containing pharmaceutical-grade extracts with high content of active principles are not recommended in the pediatric population because of the risk of interactions and side effects. At the same time, preparations that were not pharmaceutical-grade showed only partial effectiveness. Among herbal candidates for mood regulation, *C. sativus* is particularly noteworthy. Studies have demonstrated its efficacy in managing ADHD symptoms, especially as an adjunct to methylphenidate, in both children [71] and adults [164]. Moreover, although still preliminary, evidence in pediatric populations suggests possible benefits for depressive symptoms [81].

Phytotherapy remains underexplored in generalized anxiety, but evidence suggests a role in context-specific forms, such as anxiety related to medical or surgical procedures. Inhaled *L. angustifolia* essential oil is of particular interest: despite small sample sizes and variable study quality, available RCTs indicate potentially beneficial effects that justify further clinical investigation [41,42,43,44,102,107].

It is important to emphasize that the field of herbal products is highly heterogeneous from a regulatory perspective, with marked differences among pharmaceuticals, dietary supplements, and other non-pharmaceutical products regarding their clinical application. This distinction is also partially reflected in the literature reviewed. The rationale for a medicinal product is to provide a well-defined therapeutic indication, supporting its use in individuals with diagnosed conditions, while necessitating careful consideration of contraindications and clear boundaries between conventional and off-label use, including age-related considerations. In contrast, non-pharmaceutical products, particularly food supplements, are intended to promote health in otherwise healthy individuals or support physiological functions, often utilizing plants that have not yet undergone full pharmaceutical development or are aimed at broader wellness outcomes.

A fundamental question therefore arises: is our understanding of these plants sufficient to guide their rational and effective use? Beyond clinical data, preclinical studies and our original bioinformatic analyses revealed a complex pharmacological matrix. Most botanicals exhibited multitarget activity, with multiple active compounds targeting overlapping or distinct molecular networks, with high or low specificity, modulating neuroinflammation, synaptic plasticity, mitochondrial function, or neuroendocrine regulation in different modes.

For example, we found that *G. biloba* flavonoids modulate neurotransmission and growth factors and show anti-inflammatory effects, while terpene lactones act as vasoprotectors, also targeting glycine receptor interaction [76,160]. At the same time, GeneCards analysis, based on existing literature and chemical structure, revealed some targets shared by the main components of the phytocomplex linked to neuroprotection. The result is a peculiar mechanism that accounts for its wide use.

Similarly, in *H. perforatum,* flavonoids, hypericin and hyperforin have different targets, but together they have marked anti-neuroinflammatory activity, distinctive for the pharmacodynamic of this medicinal plant [165].

Some medicinal plants show more defined convergence on predicted targets. Passionflower is rich in C-glycosyl flavonoids such as vitexin, isovitexin, orientin, and isoorientin, which could also be transformed into aglycones like apigenin and luteolin. All these flavonoids could target receptors and modulate neurotransmission, decrease neuroinflammation and oxidative stress, and target MAO-A and MMPs.

Other medicinal plants such as *P. pinaster* and *M. officinalis* may exert CNS effects through a multimodal effect produced by a phytocomplex enriched in compounds that, in native form and, more likely, after metabolism, are able to cross the BBB; interestingly, according to bioinformatic prediction, these two medicinal plants may have pharmacological effects in part via MMP modulation. We acknowledge that this mechanistic insight is still speculative and requires clinical validation, but the role of MMPs, particularly MMP-9, in synaptic plasticity, neuroinflammation, and blood–brain barrier remodeling is relevant to pediatric psychiatric pathophysiology [166,167].

The fascinating, challenging, and not fully elucidated role of phytocomplexes emerges for medicinal plants such as *V. officinalis, C. sativus,* and *L. angustifolia* (essential oil), which produce pharmacological effects through complex mechanisms. The same could be said for the complex nootropic effect of *B. monnieri* and for the adaptogenic activity of *P. ginseng*.

Also, for *W. somnifera*, the mechanism of action appears to involve multiple targets shared by the constituents of the phytocomplex; however, in this medicinal plant a stronger convergence is observed toward an anti-neuroinflammatory effect, mainly mediated by its characteristic steroidal lactones.

Research into herbal products within the neuroscience field has yielded a wealth of compelling advancements in recent years, providing an opportunity to revisit and consolidate knowledge that has previously been underexplored and showcasing innovative mechanisms of action and the therapeutic potential of medicinal plants that have scarcely been investigated or whose mechanisms of action were previously poorly understood.

L-theanine, a non-proteinogenic amino acid predominantly found in green tea (*Camellia sinensis* (L.) Kuntze), has been recognized for its ability to modulate glutamatergic transmission [168]. Recent research has further elucidated its neuroprotective effects, demonstrating its capacity to attenuate neurotoxic damage and enhance cognitive functions in preclinical models [169,170]. Additionally, clinical trials have reported improvements in sleep quality and reductions in stress-related symptoms in humans [171]. However, despite these promising findings, there is a notable absence of studies specifically investigating the use of L-theanine in pediatric populations.

Emerging evidence also highlights natural products modulating the endocannabinoid system, such as β-caryophyllene [172], a sesquiterpene found in several plants including *Piper nigrum* L. or *Humulus lupulus* L., or honokiol and magnolol, found in *Magnolia officinalis* Rehder & Wilson bark (magnolia), a medicinal plant known in TCM and traditionally used for anxiety and minor mental disturbances [173]. In this case as well, studies specifically conducted in pediatric populations are currently lacking.

The need to enhance pediatric health literacy has been mentioned. Families should be empowered with accurate, evidence-based information to guide phytotherapy decisions. Clinicians must maintain open dialogue, ensuring appropriate use, monitoring, and integration into broader care plans. Finally, a critical reflection: what practical value does this accumulating evidence offer to clinicians, researchers, and policy-makers? These stakeholders must translate current findings into evidence-based guidelines, regulatory frameworks, and educational programs that ensure quality, safety, and therapeutic value. This entails stringent standardization of herbal products, well-designed pediatric trials, clear reporting of adverse events, and systematic evaluation of risk–benefit profiles. In conclusion, phytotherapy represents a promising, but still evolving, approach to pediatric neuropsychiatric care. While some botanicals are supported by clinical data, others remain experimental and require further investigation. Integration into practice must be guided by scientific rigor, regulatory clarity, and collaboration among families, clinicians, and researchers to optimize mental health outcomes in children and adolescents.

Table 6 summarizes all published studies found for pediatric mental health, organized to provide information on the clinical evidence for each medicinal plant, their preparations, their approval status as medicines or food supplements, and their use in clinical practice.

### Limitations

Although this review was planned and conducted to the best of our knowledge, this review presents limitations that should be acknowledged. First, the number and quality of clinical studies specifically conducted in the pediatric and adolescent populations remain limited. Many trials involve small sample sizes, short follow-up periods, and heterogeneous methodologies, which hinder the generalizability and strength of the conclusions. Moreover, some herbal products used in these studies are available as food supplements rather than pharmaceutical-grade preparations, often lacking detailed characterization or standardization of the phytochemical composition. Second, the regulatory status and formulation of herbal products vary widely across countries, making cross-study comparisons challenging. Age-related pharmacokinetic and pharmacodynamic differences are also insufficiently explored, and many studies do not clearly define the minimal age or developmental stage of participants, limiting the ability to draw conclusions specific to neurodevelopmental windows. Third, the bioinformatic predictions of molecular targets and mechanisms of action, while providing useful insights, are inherently limited by current databases, computational models, and the lack of experimental validation for many natural compounds. In particular, predictions may overlook the impact of metabolism, gut microbiota transformation, or the presence of conjugated metabolites, which are relevant in pediatric populations. Finally, although the integrative approach combining clinical and mechanistic evidence is a strength of this work, the lack of longitudinal, large-scale studies and the limited reporting of safety outcomes call for caution in translating these findings into clinical practice. Future research should aim to address these gaps through well-designed pediatric trials, the use of well-characterized (hopefully standardized) preparations, and mechanistic studies that take into account the unique developmental features of the pediatric brain.

## 5. Conclusions

This review highlights that, although the scientific literature on pediatric phytotherapy is still limited, a foundation for responsible clinical application and further research is emerging. A subset of medicinal plants has been investigated in randomized controlled trials, offering preliminary but encouraging evidence for specific indications in children and adolescents, whereas many other botanicals discussed remain supported by observational studies and open-label trials, warranting further investigation. Importantly, the sector of herbal products has evolved at the scientific, manufacturing, and regulatory levels, and clinicians and researchers now have access to authorized medicinal preparations and high-quality botanical ingredients, which provides a solid basis for a safer use in pediatric integrative care. Nevertheless, clinical validation remains essential. Progress in this field depends on well-designed pediatric trials, appropriate dosing and safety monitoring, and improved translational models that consider developmental and ethical specificities. Our aim is to encourage a cautious, yet scientifically grounded, expansion of evidence-based phytotherapy to support mental health and neurodevelopment in children and adolescents, anchored in current evidence but mindful of its limitations.

## Figures and Tables

**Table 1:** Clinical studies using herbal products for agitation, restlessness, and sleep disturbances in children. The regulatory status refers to the country in which the study was conducted.

Study (Author, Year)	Treatment and Regulatory Status	Study Design	Population	Treatment	Main Outcomes
Müller and Klement (2006) [38]	Valerian + lemon balm standardized dry extract (Euvegal^®^). Herbal Medicine	Multicenter observational study	918 children (6–11 years) with restlessness and dyssomnia (Germany)	4 × (160 mg valerian root dry extract + 80 mg lemon balm) tablet/day for 4 weeks	Improvement of sleep quality and reduction of restlessness according to parents’ and investigators’ assessment
Gromball et al. (2014) [39]	Valerian + lemon balm standardized dry extract (Sandrin^®^). Herbal Medicine	Multicenter observational study	169 children (6–12 years) with hyperactivity and concentration difficulties (Germany)	640 mg valerian root extract + 320 mg lemon balm extract/day for 7 weeks	Significant changes in scores related to impulsiveness, attention, hyperactivity, sleep disturbances, and social behavior according to parents and investigators
Trompetter et al. (2013) [40]	SJW + valerian + passionflower dry extracts. Combination of extracts authorized as ingredients for food supplements	Observational clinical study	115 children with nervous agitation (Germany)	60 mg SJW, 28 mg valerian, 32 mg passionflower tablet; 1–3 tablets/day	Decreased symptoms of agitation; anxiety, depression, and sleep disturbances according to parents’ and physicians’ evaluation

**Table 2:** Clinical studies of herbal products in anxiety and psychological discomfort related to procedural pain. The regulatory status refers to the country in which the study was held. RCT: randomized controlled trial.

Study (Author, Year)	Treatment and Regulatory Status	Study Design	Population	Treatment	Main Outcomes
Arslan et al. (2020) [41]	Lavender essential oil. Fragrance (not pharmaceutical-grade)	RCT	126 children (6–12 years) undergoing tooth extraction (Turkey)	Inhalation for 3 min before the intervention session	Decrease in anxiety and pain perception during dental procedures; significant decrease in blood pressure and pulse rate in the lavender oil group
Ghaderi and Solhjou (2020) [42]	Lavender essential oil. Fragrance (not pharmaceutical-grade)	RCT	24 children (7–9 years) undergoing dental treatment (Iran)	Inhalation during the intervention session	Reduction in cortisol level, pulse rate, and stress and pain perception during dental treatment
Ardahan Akgül et al. (2021) [43]	Lavender essential oil. Fragrance (not pharmaceutical-grade)	RCT, double-blind, vs. placebo	108 children (2 months–7 years) with burns undergoing dressing changes (Turkey)	Inhalation of lavender oil for 15 or 60 min prior to burn dressing change	Reduction in pain levels and stabilization of vital signs post-dressing
Soltani et al. (2013) [44]	Lavender essential oil. Fragrance (not pharmaceutical-grade)	RCT, prospective	48 children (6–12 years) undergoing tonsillectomy (Iran)	Acetaminophen (10–15 mg/kg/dose, PO) and lavender oil inhalation for 3 min or acetaminophen after tonsillectomy surgery every 6 h	Lower pain scores and reduced need for analgesics after tonsillectomy
Nord and Belew (2009) [45]	Lavender and ginger essential oils. Fragrance (not pharmaceutical-grade)	RCT, single-blind	94 children (7–17 years) undergoing surgical procedures (USA)	Inhalation during perianesthesia period	Enhanced comfort and reduced postoperative nausea

**Table 4:** Clinical studies using herbal products in depressive/anxiety disorder in children. The regulatory status refers to the country in which the study was conducted. RCT: randomized controlled trial.

Study (Author, Year)	Treatment and Regulatory Status	Study Design	Population	Treatment	Main Outcomes
Lopresti et al. (2018) [81]	Affron^®^ saffron extract (>3.5% active principles called lepticrosalides). Food supplement-grade extract	RCT, double-blind, vs. placebo	80 adolescents (12–16 years) with mild-to-moderate symptoms of anxiety/depression (Australia)	14 mg b.i.d for 8 weeks	Youth self-reports, but not parental reports, showed significant improvements in anxiety and depressive symptoms
Findling et al. (2003) [82]	SJW standardized extract. Food supplement-grade extract	Open-label pilot study	33 children and adolescents (5–16 years) with major depression (USA)	Initial dose: 450 mg/day for the first 4 weeks Dose escalation: If clinical response criteria were not met after 4 weeks, the dose was increased to 900 mg/day for the remaining 4 weeks	Clinical response, evaluated through the CDRS-R and CGI scales, was recorded after eight weeks in 25 out of 33 children

**Table 5:** Clinical studies on herbal products in ASD and dyslexia in children. The regulatory status refers to the country in which the study was conducted. RCT: randomized controlled trial.

Study (Author, Year)	Treatment and Regulatory Status	Study Design	Population	Treatment	Main Outcomes
Hasanzadeh et al. (2012) [83]	Ginkgo standardized extract (Ginko T.D.™) added to risperidone. Herbal medicine	RCT, double-blind, vs. placebo	47 children (4–12 years) with autism (Iran)	80 mg/day (<30 kg), 120 mg/day (>30 kg) with risperidone for 10 weeks	Ginkgo addition did not enhance risperidone effects in autism symptoms (ABC-C scores)
Donfrancesco et al. (2007) [84]	Ginkgo biloba standardized dry extract (EGb 761^®^). Pharmaceutical-grade ingredient	Open-label pilot trial	15 children (5–16 years) with dyslexia (Italy)	Single morning dose of 80 mg	Improvement in word list accuracy, non-word reading, and text reading; brief headache in two children

**Table 6:** Summary of medicinal plants evaluated in clinical trials for pediatric mental health: clinical evidence, preparations, and their regulatory status and pediatric use.

Botanical Name	Herbal Preparations Used in Clinical Trials in Children	Indications	Clinical Evidence and Safety Concerns	Regulatory Status and Pediatric Use
*B. monnieri*	Standardized dry extracts.	ADHD symptoms	1 RCT and 1 open-label study. Inconclusive results regarding behavioral symptoms; improvements in mood, cognition, and sleep. No safety concerns recorded for dosage and duration of treatment considered in clinical trials analyzed.	Traditional Ayurvedic remedy. Approved as food supplement ingredient in the EU and in other countries. Use: integrative/alternative medicine.
*C. sativus*	Powdered herbal material and dry extracts, chemically characterized.	ADHD symptoms and mild-moderate depression	3 RCTs and 1 non-randomized clinical trial. In ADHD: effect comparable to methylphenidate on ADHD symptoms, especially for hyperactivity symptoms, heterogenous results for inattention symptoms. In mild-moderate depression: self-reported reduction in depressive symptoms and anxiety scores. No safety concerns recorded for dosage and duration of treatment considered in clinical trials analyzed.	Used as food and as traditional remedy in many Asian countries. Powdered substance and extracts are also approved as ingredients for food supplements in the EU and in other countries. Use: integrative/alternative medicine.
*G. biloba*	Herbal medicines consisting of standardized extracts (24% flavonoids and 6–8% terpenes).	ADHD, autism, dyslexia	3 RCTs and 2 open-label pilot studies. In ADHD: reduction in ADHD parent and teacher scores, improvement in quality of life; in the comparative study, effect of *G. biloba* lower than that of methylphenidate. In autism, no additive effect for the combination *G. biloba* + risperidone compared to risperidone alone. In dyslexia, improvement in word list accuracy and reading. Mild side effects recorded after *G. biloba* preparation administration compared to placebo (headache in two children). Even if not recorded in the clinical trials analyzed, drug–drug interactions should be considered for *G. biloba,* as well as risk of bleeding and hematological concerns.	Herbal medicine authorized by the EMA in the EU in adults only. Standardized extracts, pharmaceutical-grade, are approved and registered as herbal medicines in other extra-UE countries. Off-label use in the pediatric population. *G. biloba* preparations are also available as ingredients for food supplements in integrative/alternative medicine (generally with dosage limitations or special warnings).
*H. perforatum*	Three different preparations: one prescribed standardized preparation (not specified), one extract (0.3% hypericin), and one fixed combination (60, 28, 32 mg of *H. perforatum*, *V. officinalis* and *P. incarnata* dry extracts).	ADHD symptoms; nervous agitation; major depression	1 RCT, patients with ADHD, extract 0.3% hypericin: no improvement in symptoms. 1 observational study, patients with nervous agitation, combination of *H. perforatum, P. incarnata,* and *V. officinalis* dry extracts: decrease in symptoms of agitation, anxiety, depression, and sleep disturbances. 1 open-label pilot study, patients with major depression, standardized extract under prescription: some improvement in depressive symptoms. Even if not recorded in the clinical trials analyzed, drug–drug and food–drug interactions and risk of side effects should be considered for *H. perforatum* administration, according to EMA and other international authorities documents.	*H. perforatum* preparations used in the clinical trials considered are approved as ingredients for food supplements. The use of *H. perforatum* preparations in food supplements is approved only in some countries, generally with dosage limitations and special warnings. Use: integrative/alternative medicine. In the EU. *H. perforatum* standardized preparations are mostly used as herbal medicines, approved by the EMA, and registered with full authorization indicated for adults. The use of these herbal medicines in children is off-label.
*L. angustifolia*	Essential oil (inhaled).	Procedural anxiety and stress	5 RCTs: decreased anxiety and stress and pain perception; objective outcomes recorded such as blood pressure, pulse rate, number of respirations, and cortisol level. No safety concerns recorded in the clinical trials analyzed. However, different routes of administration beside inhalation should be carefully evaluated, and oral administration of essential oils is not recommended in children < 12 years.	*L. angustifolia* essential oils used in pediatric studies are not pharmaceutical-grade, and they are marketed as fragrances or products for aromatherapy. Use: integrative/alternative medicine. *L. angustifolia* essential oil, pharmaceutical-grade, is approved by EMA as a traditional herbal medicinal product for oral use in soft capsules or as a bath additive in children over 12 years (conventional use, off-label in younger children).
*M. officinalis*	Herbal medicine consisting of a fixed combination of 2:1 *V. officinalis* and *M. officinalis* standardized dry extracts.	Restlessness, sleep disturbances, agitation, ADHD symptoms	3 observational studies combined with *V. officinalis*. Improved sleep quality and reduced restlessness; reduced hyperactivity and impulsiveness, enhanced attention. No safety concerns recorded for dosage and duration of treatment considered in clinical trials analyzed.	Herbal medicine authorized by the EMA in the EU from 6 or 12 years of age, depending on specific national authorization. Conventional use. *M. officinalis* preparations are also available as ingredients for food supplements in integrative/alternative medicine.
*P. ginseng*	Powdered herbal material (in the form of Korean red ginseng) and dry extract, chemically characterized. In one study: combination of Korean red ginseng and omega-3.	ADHD and subthreshold ADHD	2 RCTs and 1 observational study: reduction in behavioral symptoms in children with ADHD. Improvement in inattention and hyperactivity symptoms in children with subthreshold ADHD treated with Korean red ginseng and omega-3. Even if side effects were not recorded in pediatric clinical trials, the use of *P. ginseng* in children should be carefully evaluated because of risk of uncommon but plausible hormonal and other minor undesirable effects.	The two clinical trials considered are from Asia, where *P. ginseng* is officially recognized in TCM and listed in official Asian Pharmacopoeias. The preparation used in the European pediatric study is approved as a food supplement ingredient in the EU and in other countries. Use: integrative/alternative medicine. *P. ginseng* powdered herbal material, dry extract, chemically characterized, and other preparations are also available in the EU as herbal medicines, approved by the EMA, mainly as traditional herbal medicinal products only for adults. Pediatric use is off-label.
*P. incarnata*	Fixed combination (60, 28, 32 mg of *H. perforatum*, *V. officinalis* and *P. incarnata* dry extracts).	Nervous agitation	1 observational study, combined with *H. perforatum* and *V. officinalis* dry extracts. Decrease in symptoms of agitation, anxiety, depression, and sleep disturbances. No safety concerns recorded for dosage and duration of treatment considered in clinical trial analyzed.	The *P. incarnata* preparation used in the clinical trial considered is approved as an ingredient for food supplements. Use: integrative/alternative medicine. *P. incarnata* dry and liquid extracts are also available in the EU as herbal medicines, approved by the EMA, mainly as traditional herbal medicinal products, generally from 12 years of age, used in conventional medicine, off-label in younger children.
*P. pinaster*	Standardized dry extract.	ADHD	3 RCTs: increase in plasmatic glutathione levels and reduction in urinary catecholamine levels; improvement in inattention and hyperactivity symptoms. No safety concerns recorded for dosage and duration of treatment considered in clinical trials analyzed.	Approved as a food supplement ingredient in the EU and in other countries. Use: integrative/alternative medicine.
*V. officinalis*	Herbal medicine consisting of a fixed combination 2:1 *V. officinalis* and *M. officinalis* standardized dry extracts. Fixed combination (60, 28, 32 mg of *H. perforatum*, *V. officinalis,* and *P. incarnata* dry extracts).	Restlessness, sleep disturbances, agitation, ADHD symptoms	4 observational studies, 3 combined with *M. officinalis* and 1 with *H. perforatum* and *P. incarnata*: improved sleep quality and reduced restlessness; reduced hyperactivity, agitation, and impulsiveness; enhanced attention. No safety concerns recorded for dosage and duration of treatment considered in clinical trials analyzed.	Herbal medicine authorized by the EMA in the EU from 6 or 12 years of age, depending on specific national authorization. Conventional use. *V. officinalis* preparations are also available as ingredients for food supplements in integrative/alternative medicine.
*W. somnifera*	Non-standardized dry extract.	Anxiety in children with ADHD	1 RCT. Reduction in anxiety symptoms and social concerns. No safety concerns recorded for dosage and duration of treatment considered in clinical trial analyzed.	Traditional Ayurvedic remedy. Approved as a food supplement ingredient in the EU and in other countries. Use: integrative/alternative medicine.

## Data Availability

Not applicable.

## Supplementary Materials

The following supporting information can be downloaded at: https://www.mdpi.com/article/10.3390/children12091142/s1, Table S1: DSM-5 anxiety disorders features; Table S2: Herbal products approved by EMA for mental stress, mood disorders and sleep disturbances; Table S3: Summary of risk of bias and certainty of evidence (GRADE) for herbal products in pediatric mental health. GRADE certainty: ⬤⬤⬤⬤ High; ⬤⬤⬤◯ Moderate; ⬤⬤◯◯ Low; ⬤◯◯◯ Very low. Risk of bias assessed with RoB 2 (RCTs) and ROBINS-I (observational studies).; Table S4: Target prediction of the main active compounds contained in each plant species, obtained by Swiss Similarity Ensemble Approach (SEA) and Swiss Target Prediction (STP) methods, and GeneCards suite.
