# Supplementary material for: Medicinal Plants for Child Mental Health: Clinical Insights, Active Compounds, and Perspectives for Rational Use
Source: Children (Basel). 2025 Aug 28;12(9):1142. doi: 10.3390/children12091142 (PMC12468278; doi:10.3390/children12091142)
Supplement: Supplementary file 1 [file children-12-01142-s001.zip › children-3765641-supplementary.pdf]

Review

Medicinal plants for Child Mental health: Clinical Insights, Active Compounds, and perspectives for rational use

Giovanna Rigillo, Joan M.C. Blom, Arianna Cocchi, Valentina Martinucci, Francesca Favaro, Giulia Baini, Giorgio Cappellucci, Fabio Tascedda, Marco Biagi

Supplementary material

Table S1: DSM-5 anxiety disorders features

Disorder	Source of Anxiety	Common Manifestations
**Separation Anxiety Disorder**	Intense fear of being apart from attachment figures or persistent worry about events that could lead to separation	Persistent distress when anticipating or experiencing separation from attachment figures; excessive worry about potential loss; reluctance or refusal to be alone or to sleep away from home; frequent somatic complaints when separation is expected.
**Social Anxiety Disorder**	Worry about embarrassment or being negatively evaluated in social or performance situations	Marked fear of negative evaluation in social or performance situations; avoidance of speaking, participating, or interacting with peers; intense anxiety during social encounters, often leading to anticipatory worry and functional impairment.
**Panic Disorder**	Fear of recurring panic episodes or their perceived consequences	Recurrent unexpected panic attacks characterized by abrupt surges of intense fear with somatic symptoms (e.g., palpitations, shortness of breath, dizziness); persistent concern about additional attacks or their consequences; avoidance of activities or contexts associated with panic.
**Agoraphobia**	Anxiety about being in places where escape may be hard, or help may be unavailable in the event of panic-like symptoms	Anxiety about situations where escape may be difficult; frequent avoidance of crowded spaces, enclosed environments, including public transport; in severe cases, confinement to the home.
**Specific Phobia**	Strong fear related to a specific object or situation (e.g., animals, weather, needles, vehicles)	Excessive fear triggered by exposure to specific objects or situations (e.g., animals, natural environments, medical procedures); immediate anxiety, avoidance and intense distress.
**Generalized Anxiety Disorder**	Recurrent worry across multiple areas of life, often tied to a feeling of lack of control or unpredictability	Excessive and pervasive worry with loss of control across multiple domains of life; symptoms include restlessness, irritability, fatigue, muscle tension, sleep disturbances, and impaired concentration

Table S2: herbal products approved by EMA for mental stress, mood disorders and sleep disturbances.

Botanical name	Plant material used	Approved indication	Marketing authorization in the European Union
***Avena sativa* L.**	Aerial parts	Relief of mild symptoms of mental stress	Traditional use
***Crataegus monogyna* Jacq./ *Crataegus laevigata* (Poir) DC. or hybrids or related species**	Leaves and flowers	Relief of mild symptoms of stress and nervousness such as palpitations	Well established use and Traditional use
***Eschscholzia californica* Cham.**	Aerial parts	Relief of mild symptoms of mental stress and to aid sleep	Traditional use
***Humulus lupulus* L.**	Female flowers (strobils)	Relief of mild symptoms of mental stress and to aid sleep	Traditional use (also in combination with other medicinal plants). As herbal tea in the combination "species sedativae"
***Hypericum perforatum* L.**	Flowering aerial parts	Treatment of mild to moderate depressive episodes	Full authorization and Well-established use
***Lavandula angustifolia* Mill.**	Essential oil from flowers (aetheroleum). Flowers for herbal tea in "species sedativae"	Relief of mild symptoms of stress and anxiety	Mainly traditional use. As herbal tea in the combination "species sedativae"
***Leonurus cardiaca* L.**	Aerial parts	Nervous tension and nervous conditions of the heart such as palpitations	Mainly in old medicines, alone or in combination, with full authorization
***Matricaria chamomilla* L.**	Flowers	Relief of mild symptoms of stress and sleep disorders	Mainly traditional use
***Melissa officinalis* L.**	Leaves	Relief of mild symptoms of mental stress and to aid sleep	Traditional use (Full authorization for the combination *V. officinalis* dry extract/*M. officinalis* dry extract). As herbal tea in the combination "species sedativae"
***Passiflora incarnata* L.**	Aerial parts	Relief of mild symptoms of mental stress and to aid sleep	Mainly traditional use. As herbal tea in the combination "species sedativae"
***Rhodiola rosea* L.**	Root and rhizome	Temporary relief of symptoms associated with stress, such as fatigue and weakness	Traditional use
***Tilia cordata* Miller/*Tilia platyphyllos* Scop./*Tilia x vulgaris* Heyne**	Flowers	Relief of mild symptoms of mental stress	Traditional use
***Valeriana officinalis* L.**	Root and rhizome (or essential oil)	Relief of mild symptoms of mental stress and to aid sleep	Full authorization and Traditional use (also in combination with other medicinal plants). As herbal tea in the combination "species sedativae". Only traditional use for the essential oil

***Verbena triphylla* L'Hér./*Aloysia citrodora* Paláu/ *Lippia citriodora* Kunth.**	Leaves	Relief of mild symptoms of mental stress and to aid sleep	Traditional use

Table S3. Summary of risk of bias and certainty of evidence (GRADE) for herbal products in pediatric mental health. GRADE certainty: ●●●● High; ●●●○ Moderate; ●●○○ Low; ●○○○ Very low. Risk of bias assessed with RoB 2 (RCTs) and ROBINS-I (observational studies).

**Outcome / Indication**	**No. of studies, study design and (participants)**	**Treatment**	**Risk of bias (RoB2/ROBINS-I)**	**Overall certainty of evidence (GRADE)**
**Agitation, dyssomnia restlessness**	3 observational studies (~1200 children)	Herbal medicine consisting in the fixed combination of valerian and lemon balm dry extracts.<br>Combination of SJW + valerian + passionflower dry extracts.	High risk	●○○○ Very low
**Anxiety and psychological discomfort related to procedural pain**	5 RCTs, single- or double-blinded (~400 children)	Lavender oil (inhaled) (in one RCT: combination of lavender and ginger oil)	Some concerns to high risk	●●○○ Low/Moderate
**ADHD**	12 RCTs (vs placebo or methylphenidate) + 1 non-randomized clinical trial + 3 open label studies + 1 observational (~850 children)	Ginseng (powder or dry extract, alone or in combination with omega-3); French maritime pine standardized extract; withania dry extract; bacopa dry extracts; saffron (powder or dry extract); St. John's Wort dry extract; ginkgo (dry extracts, pharmaceutical-grade); herbal medicine consisting in the fixed combination of valerian and lemon balm dry extracts	Some concerns to high risk	●●○○ Low
**Depressive symptoms**	1 RCT, double blind vs placebo + 1 open-label pilot trial (~110 adolescents)	Saffron dry extract;<br>St. John's Wort dry extract	Some concerns to high risk	●○○○ Very low/Low
**Autism spectrum disorder and dyslexia**	1 RCT, double blind vs placebo + 1 open-label pilot trial (~110 adolescents) (~60 children)	Ginkgo (dry extracts, pharmaceutical-grade);	Some concerns to high risk	●○○○ Very low/Low

Table S4: Target prediction of the main active compounds contained in each plant species, obtained by Swiss Similarity Ensemble Approach (SEA) and Swiss Target Prediction (STP) methods, and GeneCards suite.

Plant species	Main constituents able to pass the blood-brain barrier	Converging targets with highest score of prediction	Probability and model accuracy of predicted targets
B. monnieri	Bacogenin A1<br>Jujubogenin	BDNF-AS, HDAC-1, HDAC-2, CB1R<br>CB2R, GPR55, NF-κB p105, CaV2.2 1B, NT-3	LOW (only from GeneCards or SuperPred)
C. sativus	Crocetin<br>Safranal	TNF-α, BDNF-AS, Catalase	LOW (only from GeneCards)
G. biloba	Quercetin<br>Kaempferol<br>Ginkgolides (mainly ginkgolide B)<br>Bilobalide	ERK2, AHR, CCL2, 5HTR3A, CREB1, α-Synuclein	LOW (only from GeneCards)
		MAO-A<br>12-LOX	MEDIUM/HIGH (from multiple tools for flavonols only)
		GLR1A<br>GLR2A	MEDIUM (from multiple tools for terpenes only)
H. perforatum	Hyperforin<br>Hypericin<br>Quercetin,<br>Amentoflavone<br>(+)-catechin and (-)-epicatechin,<br>5-(3′,4'-dihydroxyphenyl)-γ-valerolactone,<br>Caffeic acid<br>Ferulic acid	ERK 1/2, BDNF-AS, IL-8, IL-6	LOW (only from GeneCards)
		MAO-A	MEDIUM/HIGH (from multiple tools for flavonols only and from SuperPred for all compounds with the exception of hyperforin)
		GLR1A<br>VEGFA	MODERATE (from SuperPred for all compounds with the exception of hyperforin)
L. angustifolia	Linalool<br>Linalyl acetate<br>Terpinen-4-ol	TRPM8<br>NMDA2BR	LOW (only from GeneCards)
M. officinalis	Rosmarinic acid<br>Ferulic acid<br>Caffeic acid<br>Quercetin<br>Apigenin<br>Luteolin	BDNF-AS	LOW (only from GeneCards)
		MMP-1<br>MMP-9<br>5-LOX	MEDIUM/HIGH (especially MMP-9 and 5-LOX, predicted from multiple tools for hydroxycinnamic acids and flavonoids with high scores)
		MAO-A	MEDIUM/HIGH (from multiple tools for flavonoids and from SuperPred only for hydroxycinnamic acids and flavonoids)
P. ginseng	PPD<br>PPT<br>Compound K<br>ginsenoside Rg3<br>ginsenoside Rf1<br>ginsenoside Rh1	BDNF-AS, NT-3, AKT 1-2-3, mTOR	LOW (only from GeneCards)
		Butylcholinesterase, A1AR, HDAC-2, VDR, NF-κB p105, MR,<br>CB2R (and CB1R), GPR55, SCN2A	LOW (only from SuperPred)
P. incarnata	Luteolin<br>Apigenin<br>C-glycosides	CASP8, CASP9, STAT3, Nrf-2, NF-κB subunits 1, HIF-1α, BDNF-AS	LOW (only from GeneCards)

		A1AR, A2AR, ACHE, MMP-9, MMP-12, CDK5 activator 5-LOX, 15-LOX, COX-2	MEDIUM (predicted from STP and SEA for aglycones)
		MAO-A, 12-LOX	HIGH (predicted by multiple tools, also for C-glycosides in SuperPred)
		GLR1, AMPA-2, GlyT2	MODERATE (predicted only from SuperPred)
P. pinaster	(+)-catechin<br>caffeic acid<br>ferulic acid<br>5-(3',4'-dihydroxyphenyl)-γ-valerolactone	BDNF-AS, Catalase, ERK2, Nrf-2, iNOs, EGFR, HIF-1α	LOW (only from GeneCards)
		MMP1, MMP9, 5-LOX	MEDIUM/HIGH (predicted from multiple tools with high scores for hydroxycinnamic acids only)
		MAO-A, NF-κB p105, GLRA2	LOW (only from SuperPred)
V. officinalis	Valerenic acids (mainly valerenic acid)	GABAA1R, GABAA2R, GABAB1R, NF-κB p105	LOW (only from GeneCards)
W. somnifera	Withanolide A<br>Withanolide B<br>Withaferin A<br>Withanone<br>Isopellieterine<br>Anaferine<br>Cuscohygrine	AKT1, caspase-3, HSP90, BDNF-AS, SOD2-OT1, IL-6	LOW (only from GeneCards for withaferin A and withanolides)
		IκBK	MEDIUM (predicted by GeneCards and STP for steroidal lactones)
		NF-κB p105, CB2R	LOW (only from SuperPred for steroidal lactones and alkaloids)
